# SOD1 regulates CXCR4 transcription in cortical neurons for establishment of cerebral ischemic tolerance

**DOI:** 10.1016/j.jare.2025.10.039

**Published:** 2025-10-23

**Authors:** Ming-Xi Li, Guangpu Su, Ying Zhou, Zhenguo Yang, Anding Xu, Chi Kwan Tsang

**Affiliations:** a Clinical Neuroscience Institute, The First Affiliated Hospital of Jinan University, Guangzhou 510630, China; b Department of Neurology and Stroke Center, The First Affiliated Hospital of Jinan University, Guangzhou 510630, China

**Keywords:** Ischemic tolerance, Superoxide dismutase, CXCR4, Neuroprotection, Ischemic stroke

## Abstract

**Introduction:**

Understanding of cerebral ischemic tolerance could provide important insight into viable neuroprotective strategies against ischemic stroke.

**Objectives:**

To elucidate the function of superoxide dismutase 1 (SOD1) in cellular tolerance to ischemia and its underlying molecular mechanism.

**Methods:**

Two-vessel occlusion of the carotid arteries and oxygen-glucose deprivation were used for modelling global ischemic preconditioning (IPC) and *in vitro* ischemic preconditioning models, respectively. Cleavage-Under-Targets-And-Tagmentation (CUT&Tag)-sequencing analysis was performed for mapping SOD1-whole genome-binding sites in cultured cortical neurons in the *in vitro* ischemic tolerance model. Cell fractionation and immunofluorescent staining were used for analyzing the subcellular localization of SOD1 in tissue and cultured neuronal cells.

**Results:**

Here, we showed that cortex, striatum and thalamus, but not hippocampus, exhibited ischemic tolerance phenotypes after global IPC. Intriguingly, we found that SOD1 was accumulated in the cellular nucleus after IPC. Notably, cortex was the most responsive brain region in terms of neuronal SOD1 nuclear translocation in response to IPC. We further confirmed these observations in the cultured cortical neurons in the *in vitro* ischemic tolerance model. CUT&Tag-sequencing analysis revealed that SOD1 bound to promoter regions in cortical neurons, and its binding was significantly altered after IPC stimulus. Notably, SOD1 was most prominently accumulated at the gene promoter of *CXCR4* after IPC, which was correlated with transcriptional activation of *CXCR4*. Furthermore, we found that upregulation of CXCR4 expression was accompanied by modulation of gene expression resulting in anti-apoptosis. Moreover, we showed that pharmacological manipulation of CXCR4 activity could regulate cell viability through STAT3 in cortical neurons. Finally, we found that SOD1 was required for CXCR4-STAT3-mediated apoptosis in the IPC-induced ischemic tolerance.

**Conclusion:**

Therefore, these results demonstrate a non-canonical transcription-regulatory function of SOD1 which regulates CXCR4-STAT3 pathway to confer ischemic tolerance in cortical neurons.

## Highlights

• Cerebral cortex is sensitive to ischemic preconditioning (IPC)-induced tolerance.
• SOD1 rapidly translocates into the nucleus of cortical neurons after IPC treatment.
• Sod1 binds to CXCR4 gene promoter which is required for its transcriptional upregulation in cortical neurons.
• SOD1-mediated CXCR4 upregulation is neuroprotective against subsequent damaging ischemia in cortical neurons.
• CXCR4-STAT3 signaling is involved in regulation of viability in cortical neurons.

## Graphical abstract

**Figure:**
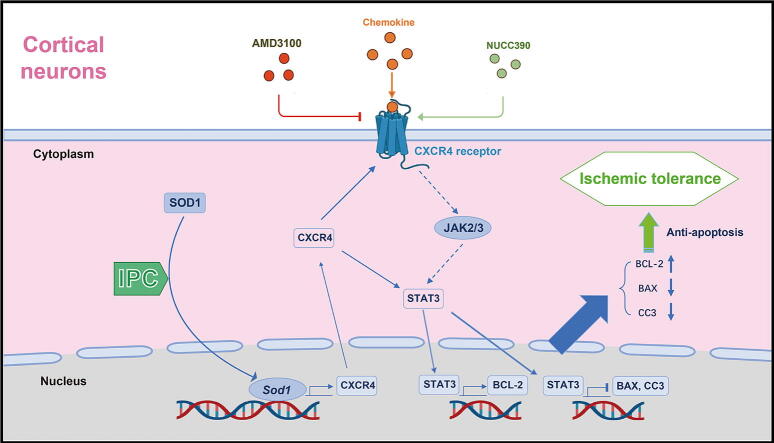

## Introduction

Cerebral ischemic stroke is characterized by high incidence of mortality and life-long disability, and accounts for the largest proportion of stroke cases worldwide among different subtypes [1]. Although recent advances have improved the reperfusion successful rate by endovascular therapies and thrombolysis after acute ischemic stroke, the therapeutic interventions for reducing stroke injuries remain limited.

The human brain has a remarkable ability to execute endogenous protective mechanisms in response to various sublethal stressful stimuli called ‘pre-conditionings’. These intrinsic defensive responses are capable of conferring significant tolerance against subsequent lethal injuries of brain ischemia such as an acute ischemic stroke. This phenomenon is referred to ‘ischemic tolerance’ [2]. In addition to mild ischemic stress, other preconditioning stimuli such as mild oxidative stress or hypoxia also exhibit protective effects on subsequent ischemic and other environmental stressors [3]. Clinical evidence such as transient ischemic attack (TIA) also revealed that brief and transient ischemic episodes occurring before severe acute ischemic stroke (AIS) is often associated with milder initial severity and favorable outcomes and prognosis of the subsequent AIS [4]. As a growing number of preclinical and clinical studies have demonstrated promising effects on improving ischemic outcomes, understanding the mechanisms underlying ischemic tolerance would provide valuable insight into the application of prophylactic treatment to those who have a higher risk of ischemic stroke as well as developing more effective and viable strategies for treatment of ischemic stroke. Indeed, manipulations of these endogenous mechanisms have been proven valuable in identifying novel and effective therapies for ischemic injury in many organs such as lung, liver, kidney and heart [5]. However, elucidation of the cerebral ischemic tolerance-associated mechanisms is still relatively unexplored.

Superoxide dismutases (SODs) are conserved antioxidant enzymes that convert superoxide into oxygen and hydrogen peroxide [6]. There are three main isoforms of SODs, namely SOD1, SOD2 and SOD3, in eukaryotic cells. SOD1 is the major form of SODs that is a copper-zine containing enzyme mainly distributes in the cytosol, mitochondrial intermembranous space, and nucleus, SOD2 is a manganese enzyme localized exclusively in the mitochondrial matrix, and SOD3 is a secretory enzyme [6]. As the first line of defense against oxidative damage, SOD1 and SOD2 function in the mitochondria to prevent excessive superoxide production during respiration, providing a direct control of the cellular level of reactive oxygen species [6]. In addition to this traditional role, emerging evidence has revealed that SOD1 can act as a regulatory protein for modulation of signaling, respiration, ribosome biogenesis and cell proliferation, playing important roles in human diseases such as cancers [[6], [7], [8]].

Recent studies have also shown that SOD1 plays a pivotal role in preconditioning stimulus-induced tolerance against subsequent injurious ischemia. However, there is still a knowledge gap about the underlying mechanism modulated by SOD1, particularly in neurons. It has been shown that low dose lipopolysaccharide-preconditioning treatment for 72 h to rats which were subsequently subjected to the middle cerebral artery occlusion (MCAO) could confer ischemic tolerance phenotype which is correlated to the brain tissue SOD1 activation [9]. Accumulating evidence has also shown that the preconditioning-induced immune responses and inflammatory reactions such as activations of cytokine production, Toll-like receptor (TLR) pathway or T-helper cells can induce ischemic tolerance [10]. It has been reported that preconditioning with TLR ligands can reprogram TLR signaling, leading to a subdued inflammatory response and enhanced neuroprotection against ischemic injury [10]. Nonetheless, it remains unclear to what extent is the role of SOD1 and immune response-mediators in modulating preconditioning-induced ischemic tolerance in the brain, particularly considering that SOD1 is an antioxidant enzyme whose expression and activity can be fluctuated in response to acute and chronic pathophysiological status. For examples, Arthur and coworkers reported that hypoxic preconditioning increases SOD1 activity in the cultured cortical neurons which is associated with the subsequent ischemic tolerance [11]. The ischemic tolerance induced by preconditioning is apparently contributed by the upregulated anti-oxidant effect of SOD1, which has been reported to provide critical function in neuroprotection against ischemic stroke surgery in animal models. Previous works by Chan *et al.* have reported that SOD1-overexpressing mice showed enhancement of the survival of neuronal cells in rat global ischemic model, while SOD1-knockdown rats exhibited enlarged infarct size after transient brain focal stroke surgery and increased neuronal loss in the cerebral penumbral tissues [12,13]. Interestingly, it has been reported that in IPC-induced tolerance rat model, neither SOD1 activity nor its protein expression is changed, yet ischemic tolerance is still induced [14]. In agreement with these observations, our previous study using oxidative preconditioning in the budding yeast as a model of preconditioning-induced tolerance also showed that SOD1 indeed exerts a crucial function in establishing the tolerance phenotype induced by preconditioning stimulus but the preconditioning *per se* does not change the activity and expression of SOD1 [15]. Therefore, these observations from us and others suggest that SOD1 confers preconditioning-induced tolerance through SOD1′s atypical function in addition to its canonical superoxide dismutase activity. Despite the recognized significance of immune responses in brain ischemia, a critical gap exists in understanding how fluctuations in SOD1 specifically interact with these immune pathways to influence ischemic preconditioning-induced tolerance.

In the present study, we employed the bilateral occlusion of common carotid artery (or two-vessel occlusion, 2VO) in C57BL/6 mice as the transient global ischemic model because it is a well-established model that takes advantage of the specific dysplasia structure of the posterior communicating artery in C57BL/6 mice in which different degrees of ischemic stimulus in different parts of the brain can be induced [16]. By using the 2VO-ischemic preconditioning model, we firstly identified the brain region that exhibited the most prominent IPC-induced ischemic tolerance. Then, we explored in different cerebral regions how IPC induced the protective effect on neurons, and characterized SOD1′s role in IPC-induced ischemic tolerance. We discovered the transcription-regulatory function of SOD1 in cortical neurons which mediates the activation of CXCR4-STAT3 pathway for anti-apoptotic mechanism. These results lead to the identification of novel targets which possess potential for effective treatment for ischemic stroke.

## Material and methods

### Experimental animals

All mouse experiments were conducted in accordance with the ARRIVE guidelines (v 2.0), and the care and use of laboratory animals were strictly complied with the National Institutes of Health guidelines (NIH Publications No. 8023, revised 1978). The ethical policies and procedures were approved by the Ethics Committee of the Animal Experimentation of Jinan University. The approval number for this study is #2019285. Female C57BL/6 timed pregnancy mice were purchased from the Chinese Academy of Medical Science Institute (Laboratory Animal), Guangzhou, China. These pregnancy mice were used for isolation of primary cortical neurons. Adult male C57BL/6 mice (6–8 weeks of age and weighed 22–28 g) were purchased from the Chinese Academy of Medical Science Institute (Laboratory Animal), Guangzhou, China, and they were mainly used as the stroke models and other experiments. They were housed in a 12h light–dark cycle daily with maintenance of rearing temperature at approximately 23 °C. The relative humidity was controlled between 40% and 60%. Animals were allowed to access food and water freely in their own cages at all times. Randomization was used for grouping and selection of all experimental animals in procedures including stroke surgeries and drug treatments. For this purpose, computer-generated random numbers were used to designate the identity of animals.

### Isolation of cultured cortical neurons

The cultured cortical neurons were collected by the isolation procedure of primary cortical neurons as described in our previous study [17]. Pregnant mice were used for isolation of embryos in which the primary cortical neurons were collected from the embryonic brains. Briefly, 3–4 months old C57 BL/6 timed pregnant mice (approximately 25 – 30 g) were anesthetized by 4% isoflurane, and embryos at E16.5 stage were collected. The heads of the embryos were carefully cut out and the primary cortical neurons were isolated from the cerebral cortex of the brains. To reduce the time of animal suffering during the procedure, cervical dislocation was performed on the mice before dissection when the anesthetic took effect within 5 min. Seven embryos could be usually obtained per pregnant mouse. Embryos were discarded if they showed fetal abnormalities such as abnormally small or big size, or deformed brain structures. The resulting cortical neurons were pooled before plating for each experiment. Neurobasal medium supplemented with 2% B27 and 2 mM glutamine (Thermo-Scientifics) was used for culturing the cortical neurons at 37 °C in a 5% CO_2_-containing humidified atmosphere in a tissue culture incubator. For cell-based experiments using the cultured cortical neurons, the unique codes were assigned to control and experimental groups for masking the sample identities. For the sample size estimation, Resource Equation methods and Power Analysis were adopted as described previously [18,19]. Standardized effect size for 90% power was used. No exclusion criteria were predetermined.

### Reagents and antibodies

Penicillin and Streptomycin (pen-strep, Biological Industries, CAS: 03–031-1B), (DMEM basic (1X) Medium, Gibco, CAS: C11995500BT), DNase I (Deoxyribonuclease I from bovine pancreas, Sigma, CAS: D4513-1VL), DMEM/F12 medium (Gibco, CAS: C11330500BT), Fetal Bovine Serum (FBS, Thermo, CAS: 10270–106), poly-lysine (PLL, Poly-L-lysine hydrobromide, Sigma, P1274-100MG), Neurobasal medium (Thermo, CAS: 21103–049), glucose-free DMEM (PM150270, Procell), AMD3100 (Merck, CAS: 155148–31-5), NUCC-390 (Glpbio, CAS: GC39573), polyformaldehyde (PFA, ServiceBio, G1101), Nissl staining solution (Beyotime, CAS: C0117), Neutral balsam (Dalian Meilin Biological Technology, LOT S0813A), lysis buffer (RIPA, Beyotime, P0013K, 1 mmol/L PMSF). NUCC-390 and AMD3100 were selected to respectively activate and inhibit CXCR4 in this study due to their reported specificity and potency on CXCR4 [20]. Primary antibodies used in this study included β-actin (Cell Signaling Technology (CST), #4970S; Beyotime, AF0003), BAX (CST, #2772), BCL-2 (CST, #3498), β-Tubulin (CST, #2146), GAPDH (CST, #9023), cleaved caspase-3 (CST, #9661), H3K27ac (#8173, CST), Lamin B1 (CST, #13435), NeuN antibody (Sigma-Aldrich, MAB377), normal rabbit IgG (#2729, CST), Rpb1 (#14958, CST), SOD1 (ab16831, Abcam; Santa Cruz, sc-17767; Sigma-Aldrich, YA3541), STAT3 (CST, #9139), and secondary antibodies included goat anti-rabbit IgG (ab6702, Abcam; Beyotime, A0208), goat anti-mouse IgG-HRP (Beyotime, A0216), goat anti-mouse IgG-AF350 (Beyotime, A0412), donkey anti-rabbit IgG-AF555 (Beyotime, A0453).

### Global cerebral ischemic surgery

**Fig. 1:**
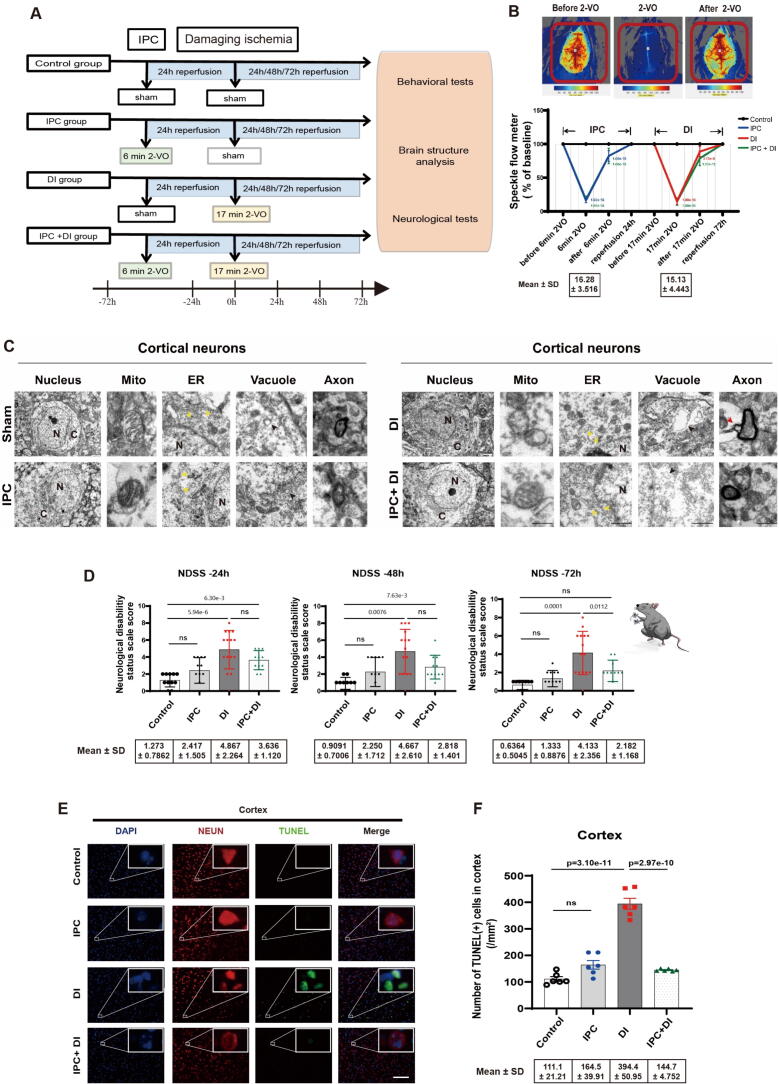
**Ischemic preconditioning (IPC)-induced tolerance in the global cerebral ischemic model. (A)** Experimental scheme for the mouse 2VO global ischemia model. Male mice were subjected to ischemic preconditioning (IPC) and/or damaging ischemia (DI) by the two-vessel (2VO) surgical procedure. **(B)** Representative images showing the blood flow monitored by the Laser Speckle Contrast Imaging System in the brain of experimental stroke mouse model. Red and blue colors indicate the brain regions with high and low levels of blood flow, respectively. Lower panel indicates the quantification of blood flow reduction compared with basal levels during the IPC and DI treatments. **(C)** Transmission electron microscopic images of cortical neurons in sham and different ischemic treatment groups. The ultrastructure of major organelles including the nucleus (N), cytoplasm (C), mitochondria (Mito), endoplasmic reticulum (ER), vacuoles and axons are shown. Representative injuries include swollen mitochondria with disrupted internal cristae, distension of ER (yellow arrowheads), enlarged vacuole (black arrowheads), and axons with thinner and demyelination-like structure (red arrowhead). Scale bar = 2 μm. **(D)** Neurological motor function was determined by the Neurological Disability Status Score (NDSS) measured at 24 h, 48 h, and 72 h after the 17 min 2VO surgery. Error bars indicate mean ± SD, n = 9 per group. Statistical analysis was performed using one-way ANOVA coupled with Tukey's multiple comparisons test; *p* values are shown on top of the bars; ns, no significant difference. **(E)** TUNEL immunofluorescent staining of the cortex in the indicated treatment groups. Magnified images are shown in the inset boxes. Scale bar = 100 μm. **(F)** Quantification result of the TUNEL-stained cells in (E). Error bars indicate mean ± SD, n = 6 per group. Statistical analysis was performed using one-way analysis of variance (ANOVA) with Tukey’s multiple comparison test; *p* values are indicated above the bars; ns, no significant difference.

Surgeries of two-vessel occlusion (2VO) of the carotid arteries were used for modelling global cerebral ischemia as described previously [16]. Briefly, C57 BL/6 mice were anaesthetized, incised on both sides of the neck. The common carotid arteries were then carefully pulled out, and tied with a 4/0 silk thread loosely for enabling subsequent occlusion of the blood vessel with an aneurysm clipping device. Mouse cerebral blood flow was monitored by the Laser Speckle Contrast Imaging System (PeriCam PSI System, Perimed AB, Stockholm) to ensure successful blood vessel occlusion after the ischemic surgery. Throughout the surgery, the mice were placed on a heating pad and the rectal temperature was monitored and ensured to be maintained at 37 °C to avoid hypothermia. After the surgery, the mouse head skin was immediately sutured before transferring them back to their own cages. The occlusion of 2VO for 6 min and 17 min were used for ischemic preconditioning and damaging ischemia, respectively [[21], [22], [23]]. For sham operation, all of the above surgical procedure was performed excepted the occlusion step, which served as control to compare the effect of ischemic treatment. The schematic diagram for the typical *in vivo* ischemic tolerance experiment is illustrated in Fig. 1A.

### Oxygen-glucose deprivation

The cell-based ischemic model using oxygen-glucose deprivation (OGD) were conducted following the method as previously described [17] with minor modifications. Briefly, the cultured cortical neurons were rinsed with phosphate-buffered saline (PBS) solution for two times. The cells were then incubated in the OGD medium which was designated as deoxygenated glucose-free Dulbecco's Modified Eagle Medium (DMEM) prewarmed at 37 °C) in the DG250 Anaerobic Workstation (Don Whitley Scientific) filling with gas mixture of 85% nitrogen, 10% hydrogen, 5% carbon dioxide for the desired length of time. The OGD medium was deoxygenated in the Anaerobic Workstation for at least 3 h before the ischemic treatment. To terminate OGD treatment, the cell culture plates were removed from the Anaerobic Workstation and the OGD medium was immediately replaced by the normal culture medium before transferring back to regular normal cell culture incubator in normal conditions. The parallel control was set up by following the same experimental procedure except that the OGD incubation was replaced by normoxic incubation in normal culture medium. OGD treatment for 30 min and 4h were designated as the ischemic preconditioning stimulus and injurious ischemia, respectively. Culture in reoxygenated and normoxic conditions were used as “reperfusion” after OGD treatments. The schematic diagram for the typical *in vitro* ischemic tolerance is illustrated in Fig. 3A.

### Cleavage-Under-Targets-and-Tagmentation and bioinformatic analyses

Cleavage-Under-Target and Tagmentation (CUT&Tag) analysis was conducted following the method as described in our previous study [24]. Briefly, freshly prepared 1% paraformaldehyde solution was used to fix the neurons at the time of sample collection. After fixation at room temperature for 5 min, 125 mM glycine solution was immediately added to the fixed cells which were further incubated in 4 °C for five more minutes. Then, the Wash Buffer containing 20 mM HEPES at pH 7.5, NaCl at 250 mM, spermidine at 0.5 mM, supplemented with protease inhibitor cocktails (Roche cOmplete, Mini, EDTA-free Protease Inhibitor Cocktail Tablets, cat. # 11,836,170 001) was used to rinse the cells gently at room temperature. After that, the cells were incubated in the Antibody Buffer containing NaCl at 150 mM, spermidine at 0.5 mM, 20 mM HEPES at pH 7.5, 0.05% Digitonin, 0.1% BSA, protease inhibitor cocktail (Roche, cat. # 11,836,170 001) and 2 mM EDTA. The corresponding antibodies and appropriated amount of ConA beads were then added to the cells according to the instruction manual. Primary antibodies used in the CUT&Tag experiment included: Rpb1 (1:100 dilution, #14958, CST), H3K27ac (1:200, #8173, CST), SOD1 (1:50 dilution, ab16831, Abcam), and normal rabbit IgG (1:50 dilution, #2729, CST). For the primary antibody incubation, cells were incubated in the Antibody Buffer containing 150 mM NaCl, 20 mM HEPES pH 7.5, 0.5 mM spermidine, 2 mM EDTA, 0.05% digitonin, 0.1% BSA, protease inhibitor cocktail (Roche, cat. # 11,836,170 001) at 4 °C for 12 h with slow-speed rotation motion on a rack. The secondary antibody of goat anti-rabbit IgG (ab6702, Abcam) with the dilution of 1:200 was used in Dig-Wash Buffer which contained 150 mM NaCl, 20 mM HEPES pH 7.5, 0.5 mM spermidine, protease inhibitor cocktail (Roche, cat. # 11,836,170 001), 0.05% digitonin). Cells were incubated in the secondary antibody at room temperature with gentle rotation motion. After one-hour incubation, cells were then rinsed with digitonin-containing wash buffer for three times in Dig-300 buffer containing 300 mM NaCl, 20 mM HEPES pH 7.5, 0.5 mM spermidine, protease inhibitor cocktail (Roche, cat. # 11,836,170 001), and 0.01% digitonin. After final wash, cells were incubated in the same buffer for one hour at room temperature. The step of transposome treatment and tagmentation procedure were performed based on the user manual of the CUT&Tag Kit (Novoprotein Scientific, China, catalog #N259-YH01). To perform the de-crosslinking step, proteinase K was added to the cells and incubated at 50 °C for one hour. DNA de-crosslinking was continued by incubation in 1% SDS in the same buffer at 65 °C for 8 h. After de-crosslinking, DNA was purified using MinElute columns (Qiagen 28006). PCRs were set up using the specific combination of indexed i5 and i7 primers in the master mix solution containing NEB Next HiFi 2x PCR reagents according to the user manual of the CUT&Tag Kit for the construction of DNA libraries. PCR conditions: step one: 72 °C for 5 min; step two: 98 °C for 30 s; step three: 98 °C for 10 s; step four: 63 °C for 30 s; step five: 72 °C for 1 min; step six: repeated 10–25 cycles from step three to step five; step seven: 72 for 2 min. Library DNAs were then purified again by the MinElute columns (Qiagen 28006) as described previously. Qubit and the corresponding super sensitive detection reagents were used to quantify the DNA amount and for determination of DNA quality. The resulting DNA fragment sizes were determined by Agilent Bioanalyzer, and NovaSeq 6000 system (Illumina) was used for the pair-end DNA sequencing with a depth of 16 million reads (Novogene, Beijing). FASTQ files were obtained by Standard Illumina software for evaluation of data quality. Bioinformatics analyses of the CUT&Tag-sequencing results were essentially performed as described in our previous study [17]. Briefly, the raw sequence reads were trimmed using PICARD, and they were aligned to the mouse reference genome (mm10) with Bowtie2 software package (http://bowtie-bio.sourceforge.net/bowtie2/). Binding peaks, merged peak sets and the differential binding peaks between experimental groups were determined by MACS2. For visualization of the binding peaks at the genome, IGV genome browser was used. The Database for Annotation, Visualization, and Integrated Discovery (DAVID) and the Genomic Regions Enrichment of Annotations Tool (GREAT) were used for performing functional annotations including Gene Ontology (GO), Kyoto Encyclopedia of Genes, and Genomes (KEGG) and BioCarter pathway analyses, and significance of cis- regulatory regions identified by the DNA-binding events across the entire mouse genome. Annotation of the binding peaks and motif analysis of the binding sequences were performed using the HOMER package in the mouse genomic background. *P* values < 0.05 were considered significant for the enrichment of these biological processes and pathways.

### Behavioral tests

Behavioral tests used in this study included NDSS, adhesive-removal, open-field, and front paw-pulling tests. All experimental animals were subjected to pre-training for determination of baseline performance before surgery. Behavioral tests were performed at 24h, 48h, and 72h after the damaging ischemic or the corresponding sham surgery. Before starting behavioral tests, animals were placed in the test environment for at least 1 h for adaptation to the experimental environment. All behavioral tests were conducted in quiet room to avoid environmental interference. Evaluation of all behavioral tests were performed by investigators blinded to the experimental treatments and animal groupings to avoid bias.

### NDSS neurological score

Mouse sensorimotor function was determined by the neurological deficit status scale (NDSS) [25]. Zero-point represents normal sensorimotor function; 2-point represents a slight neurological deficit, 4-point represents a moderate neurological deficit, such as mild slow-moving, slow-moving posture, gait disorder, erection of hair, muscle weakness, decreased vocalization, and mild dyskinesia; 6-point represents severe neurological deficit, with moderate to slow moving capability, circling motion, tremor, decreased forepaw pull, moderate dyskinesia; 8-point represents severe dyskinesia, dyspnea, inability to move; 10-point represents death.

### Adhesive removal test

Sensory and motor coordinated neurological functions were determined by Adhesive Removal test with minor modifications [24]. Specifically, the experimental mouse was placed inside the assessment box for an hour for acclimatization of the testing environment. To start the assessment, an adhesive tape (1 cm × 1 cm) was pasted on the mouse’s front paws in the assessment box. The time taken for the mouse to sense the adhesive tape on their paws and the time taken for them to start scratching the tape with its mouth or front paw was designated as the sensory functional deficit of the motor cortex [26]. For the correlated proxy assessment of coordination of sensory and motor function by the thalamus, the time the mouse taken for sensing the tape and complete removal of the adhesive tape from its limb was measured. The baseline value of the test was determined by performing the test for three consecutive days before surgery [27].

### Open field test

The mouse striatal functional assessment was conducted by the modified Open field test (OFT) as described [[28], [29], [30]]. Briefly, the mouse was positioned at the fixed corner point on an OFT platform with a dimension of 90 cm × 90 cm (Shanghai Xinsoft Instrument Co., Ltd.). The mouse's initial movement trajectory and total steps were recorded by the OFT-equipped digital video camera for a period of five minutes. The movement trajectory by OFT-analyzing software was used as a proxy correlated assessment for striatal function. The open field test was conducted at approximately the same time in the morning for three consecutive days.

### Hanging wire test

Hanging wire test is based on the latency of a mouse to fall off a metal wire and allows assessment of motor neuromuscular impairment and coordinated movement capacity of front paws that is correlated with striatal function [31]. The apparatus was consisted of a single 2 mm in diameter wire that was mounted between two ring stands with 37 cm height. A cotton padding was placed under the wire between the ring stands to avoid animal injury. By holding the tail, a mouse was allowed to grasp the middle of a 55 cm in width and 2 mm in diameter metallic wire with its fore limbs only. The tail was released while the mouse was still grasping the wire with its fore paws. Upon release, a timer was started, the time until the mouse completely released its grasp and fell down was recorded. The latency to fall was measured five times for each mouse. The first two trials served as pre-training, and the subsequent three trials were taken at ten-minute intervals, and the average time of latency to fall was recorded. No attempt was made to force compliance during any of the various trials. If the mouse adopted a strategy that permitted an extended hanging time, this was allowed and the true latency to fall time was recorded. If a mouse was unable to hang from the wire for less than one second, a fall time of zero was noted for that trial.

### Front paw-pulling test

The muscle strength of experimental mouse’s front limbs as well as its body balance ability were assessed by the front paw-pulling test. The mouse was allowed to grasp a rope at the middle position with both of its paws at a height of 40 cm above the ground. The time before it dropped down from the rope to pad inside the assessment device was recorded. Before each assessment, the mouse had been placed in the assessment case for one hour for adaptation of the experimental environment. Pre-training was conducted at the same time in the afternoon for three continuous days before the surgery for obtaining the baseline values for each mouse. After surgery, the anterior paw tensile testing was conducted as the pre-training sessions.

### Transmission electron microscopy

The ultrastructure of the cortical and hippocampal neurons were analyzed by transmission electron microscopy as described previously with minor modification [32]. Briefly, brain tissues were fixed with 2.5% glutaraldehyde and 1% osmium tetroxide in 0.1 M phosphate-buffered saline (pH 7.4) at 25 °C for 2 h. After being dehydrated in graded ethanol (50%, 70%, 80%, 90%, 95%, 100%), the samples were embedded in 1:1 mixture of acetone and Embed-812 agent overnight, and then incubated at 60 °C for 48 h. Samples were then cut into 70-nm-thick sections and stained with uranyl acetate and lead citrate. The neuronal structures were imaged using an electron microscope (FEI TecnaiG2 20 TWIN).

### Propidium iodide staining

Propidium iodide (PI) staining method was used to determine the cell viability [17]. The isolated live neuronal cells were exposed to the PI staining solution (7 μM in phosphate-buffered saline; excitation maximum for PI is 535 nm and the emission maximum) for 15 min. The residual staining solution was removed by rinsing cells for three times with PBS. Then, the cells were observed under a fluorescent microscope with an excitation filter set of 535–555 nm, and the number of PI-positively stained cells were recorded with an emission filter set of 610–630 nm. The ratio of PI-positive cells over the total number of cells was determined. For each well of the culture dish, at least three fields of view were counted per biological replicate and image J was used for quantification.

### Trypan blue staining

Cell death was determined by the standard trypan blue staining method as described [33]. The cells which were stained light blue were considered as dead cells while cells without prominent blue staining were regarded as surviving cells. For the assessment, cells were incubated in 0.04% trypan blue reagent for 5 min, and after rinsing the cells for three times with PBS, they were counted under an optical microscope. ImageJ was used for quantification of dead cells, and at least 3 fields of view were taken for each well of the culture dish per biological replicate.

### Nissl staining

Nissl staining was used to evaluate the lesion of neurons in the affected brain regions [24]. Briefly, the frozen brain samples were fixed with 4% paraformaldehyde (PFA), washed three times with distilled water. Brain slices were then stained with Nissl staining working solution for ten minutes at room temperature. After washing with distilled water for two times, slices were undergone dehydration process in 95% ethyl alcohol and then followed by 70% ethyl alcohol. After two more washings, the slices were incubated in xylene solution for 5 min, and fresh xylene solution was used for incubation for 5 min more. Then, neutral resin was used to seal the slices which were then observed under an optical microscope. Injured neuronal cells were identified by abnormal Nissl body morphology with dark blue color, whereas normal neuronal cells were stained with regular mottled Nissl bodies with bluish purple color.

### Indirect immunofluorescent staining

Indirect immunofluorescence (IF) analysis was used for visualization of SOD1 in cultured cortical neurons. Cells were seeded on a glass coverslip coated with poly-L-lysine (PLL) on a 24-well plate at a cell density of 0.2×10^6^ cells per well. Cells were fixed by incubation in 4% formaldehyde in PBS for 15 min. Cells were then incubated in primary and secondary antibodies as described before [24]. For staining of nucleus, cells were stained with 50 ng/ml 4′,6-diamidino-2-phenylindole (DAPI, excitation maximum at 358 nm and emission maximum at 461 nm) for 15 min at room temperature. Fluorescent images of the cells with localization and intensity of various fluorescent signals were analyzed using a confocal fluorescent microscope with the appropriate excitation and emission filter sets. The excitation filter set of 340–380 nm and emission filter set of 420–470 nm were used for blue fluorescence. The excitation filter set of 450–490 nm and emission filter set of 500–550 nm were used for green fluorescence, whereas the excitation filter set of 510–560 nm and emission filter set of 600–660 nm were used for red fluorescence. Quantitative and statistical analyses for the percentage of cells showing nuclear localization or fluorescent intensity were performed as described previously [24].

For indirect immunofluorescent analysis of the brain slices for visualization of SOD1 and neurons, the paraffin-embedded sections were rehydrated and then incubated in the antigen retrieval solution as described [24]. Briefly, the rehydrated slices were microwaved for five minutes at high power level and five minutes at middle power level in a regular microwave oven. After one hour cooling down at room temperature. Slices were incubated in Repair Solution and rinsed for two times with PBS (ten minutes each time), incubated with 5% goat serum containing the primary antibodies for 1.5 h at room temperature or overnight at 4°C. Mouse NeuN antibody (1:400), rabbit NeuN antibody (1:400), and rabbit SOD1 antibody (1:400) were used as primary antibodies in this study. Corresponding secondary antibodies were then incubated for one hour to three hours at room temperature. Sealing solution containing DAPI (50 ng/ml) was used for sealing and staining of the nuclei. Images were acquired using a confocal or fluorescence microscope. ImageJ software was used for the purpose of image processing and quantification of the fluorescent signals and cellular morphologies. All experimental procedures and quantitative analyses were carried out by researchers who were blinded to the experimental treatments and group identities.

### TUNEL staining of apoptotic cells

Determination of brain damage was performed by TUNEL as described [4]. Briefly, TUNEL-labelled cells were determined by the one-step TUNEL detection kit (C1086, Beyotime Biotechnology, China) and visualized by a fluorescent microscope with an excitation filter set of 450–490 nm and emission filter set of 500–550 nm. Neurons were stained with NeuN antibody as described above. Nucleus were counterstained by DAPI (Life Technologies, Mulgrave, VIC, Australia) for five minutes at room temperature. Slides were finally sealed with quenching solution containing anti-fluorescence agent, and images were obtained with an inverted fluorescence microscope (Leica, Wetzlar, Germany).

### Cell fractionation, protein extraction, and Western blot

For separation of proteins in the cytoplasm and nucleus, the Nuclear and Cytoplasmic Protein Extraction Kit was used following the user instruction manual (Beyotime, P0027), and the extraction of proteins from separated nuclear and cytoplasmic fractions was performed according to the Western blot analysis as described [24]. Briefly, cultured neuronal cells or the corresponding portion of the brain tissues (cortex, striatum, thalamus and hippocampus) were subjected to protein extraction in RIPA lysis buffer (50 mM Tris-HCl (pH 7.2), 150 mM NaCl, 1% NP-40, 2 mM EDTA, 0.1 mM PMSF, 0.1% SDS, protease and phosphatase inhibitor cocktails (Roche, cat. # 11,836,170 001)). Protein samples were then loaded onto 8–12% SDS-PAGE, followed by transferring to PVDF membrane (Bio-Rad). Membranes were blocked in Tris-buffered saline Tween (TBST) supplemented with 5% non-fat dry milk for one hour at 25 °C or 4 °C overnight. After blocking, membranes were incubated in the fresh blocking buffer containing the corresponding specific primary antibodies for 1h at 25 °C or 4 °C overnight. The appropriate dilution for each primary antibody was determined according to the manufacturer’s recommendations or empirically, as followed: SOD1 antibody (Santa Cruz, sc-17767, 1:1,000), β-actin (CST, #4970S, 1:1,000) (Beyotime, AF0003, 1:1,000), GAPDH (CST, #9023, 1:1,000), Lamin B1 (CST, #13435, 1:1,000), STAT3 (CST, #9139, 1:1,000), BCL-2 (CST, #3498, 1:1,000), 1:1,000), Cleaved Caspase-3 (CST, #9661, 1:1,000), BAX (CST, #2772, β-tubulin (CST, #2146, 1:1,000). Membranes were then incubated in TBST containing 5% milk and the corresponding horse radish peroxidase-conjugated secondary antibodies. The resolved protein bands were visualized and analyzed with the Gel Imaging System (Tanon 2500), and quantification was performed by Quantity-One (Bio-Rad) or ImageJ software. The commonly used loading controls including GAPDH, β-actin or β-tubulin were used for normalization of sample loadings.

### RNA extraction and RT-qPCR

For brain tissues, samples were homogenized with a tissue grinder Tissuelser-24, (Shanghai Jingxin Industrial Development Co., Ltd., Cat # 016518382) at 4 °C using a power output of 70 Hz for 90 s. TRIzol reagent (Thermo Fisher, CAS: 15596026) was used for extraction of RNA as described [24] with minor modifications. Briefly, after RNAs were extracted from the cultured cortical neurons or from samples of the homogenized mouse brain tissues, the reverse transcription (RT) step was performed according to the instruction manual of Evo M−MLV RT Mix kit (AG 11728), and quantitative real time PCR (qPCR) was performed using the SYBR Green Pro Taq HS qPCR kit (AG11701). In addition, SYBR Green Real-time PCR Master Mix (NEB, CAS: M0544S) was used for PCR amplification of cDNA. At least three technical replicates per sample were used for setting up the reactions using the SYBR Green Pro Taq HS qPCR kit (AG11701). For PCR program, one hold at 95 °C for 1 min, followed by 40 cycles of 95 °C (5 s), 60 °C (30 s), and 95 °C (10 s) was used for the thermocycling reactions. The melting curve analysis was used for ensuring that all of the PCR products were specific, and contained no contamination and primer dimmers. Messenger RNA levels of the house keeping genes *GAPDH* or *ACT1* were used for the normalization purpose. Relative RNA levels were determined by standard curves method. The primer sequences used for PCR in this study can be found in Supplementary Table S1.

### Statistical analysis

Statistical Package for the Social Sciences (version 27.0; SPSS Inc., Chicago) and GraphPad Prism 8.4.0 statistical software were used for statistical analysis in this study. In general, the Shapiro-Wilk test was used for normality analysis of datasets. For data with Shapiro-Wilk *p* > 0.05, the parametric tests including student’s t-tests (two-tailed), paired *t*-test or one-way ANOVA were performed. FISHER'S (Least Significant Difference, LSD) test was also used for pairwise comparisons between multiple samples, whereas Tamhane's T2 test was used for unequal variances. For data with Shapiro-Wilk *p* < 0.05 or for experiments with sample size n < 6, the Wilcoxon Mann-Whitney test or Wilcoxon matched-pairs signed rank test was used as non-parametric test. *P* values < 0.05 were regarded as having statistically significant difference. For multiple comparison, we used Tukey’s multiple comparison test to adjust the *p* values. The robust regression and outlier removal method (ROUT) with a Q = 1% false discovery rate in GraphPad Prism was used for identification of outlier in datasets. With respect to the bioinformatic analyses including DAVID, GO and KEGG, *p* values < 0.05 were considered significant for the enrichment of these biological processes and pathways.

## Results

To explore the potential role of SOD1 in regulating ischemic tolerance in neurons, we used the two-vessel occlusion (2VO) of bilateral common carotid arteries for the global transient ischemic tolerance model because this is a well-established model that allows the same surgical procedure for achieving both ischemic preconditioning and damaging ischemia, and allows simultaneous examination and comparison of multiple brain regions per sample. To select the appropriate brain region for downstream studies, we first compared the ischemic tolerance capability in the major brain regions including the cortex, striatum, thalamus and hippocampus using the 2VO mouse model.

### Transient global ischemia induces prominent ischemic tolerance in the cerebral cortex

According to the literature and our preliminary results, we adopted a 6 min-2VO and a 17 min-2VO to induce ischemic preconditioning (IPC) and damaging ischemia (DI), respectively [[21], [22], [23]]. We set up four experimental groups, including the sham control group, IPC only group, damaging ischemia group, and IPC + DI group, as depicted in the experimental scheme in Fig. 1A. Successful occlusion of the carotid arteries by the 2VO surgery in mice was confirmed by reduction of the blood flow in the brain using the Laser Speckle Contrast Imaging System (6 min 2VO: 16.28 ± 3.516%, *p* = 1×10^-15^; 17 min 2VO: 15.13 ± 4.443%, *p* = 1×10^-15^, Fig. 1B). We used Nissl-immunohistochemistry staining and NeuN-immunofluorescent staining to determine the brain damage 72h after the damaging ischemia surgery. For the cerebral cortex, we examined the motor cortex zone 1 (M1), motor cortex zone 2 (M2) and sensory cortex (S1). As expected, 6 min-2VO IPC did not cause any significant damage in all tested cortical regions compared with the sham control (control vs IPC, Supplementary Fig. S1A-D). In contrast, 17 min-2VO caused significant neural injury as revealed by prominent neuronal loss (control vs DI, Supplementary Fig. S1A-D). Notably, pretreatment with IPC significantly alleviated the neuronal damage imposed by the subsequent 17 min-2VO treatment in the M1, M2, and S1 regions (M2: 291.7 ± 98.95 vs. 497.9 ± 67.74, *p* = 5.07×10^-4^; M1: 293.8 ± vs. 458.3 ± 87.20, *p* = 0.0038; S1: 345.8 ± 85.76 vs. 520.8 ± 137.5, *p* = 0.0285 (DI vs. IPC+DI, Supplementary Fig. S1B), Supplementary Fig. S1D). We also used transmission electron microscopy (TEM) to analyze the pathological features of IPC-induced tolerance. As shown in Fig. 1C, cortical neurons in sham control and IPC-only group exhibited normal ultrastructure of major organelles including mitochondria, endoplasmic reticulum (ER), nucleus and axons (Fig. 1C, left panel). In contrast, the DI group showed prominent neuronal injuries including deformed nucleus, swollen mitochondria with disrupted internal cristae, distension of ER, enlarged vacuoles, and axons with thinner and demyelination-like structure (Fig. 1C, right panel, DI group). Consistently, we observed that IPC pretreatment alleviated the DI-induced damages in these structures (Fig. 1C, right panel, IPC+DI group). To verify whether the structural protection offered by IPC against subsequent damaging ischemia-induced injury on these brain tissues was correlated with the corresponding motor deficits, we used the Neurological Disability Status Score (NDSS) to evaluate the neurological functions in the motor cortex in which higher scores indicate more severe neurological motor deficit [25]. As shown in Fig. 1D, the NDSS were significantly higher in the 17 min-2VO group at 24h, 48h, and 72h after the damaging ischemia surgery compared with sham controls. However, IPC + DI group exhibited significantly improved neurological function compared with the DI-only group (NDSS-72h: 4.133 ± 2.356 (DI) vs. 2.182 ± 1.168 (IPC + DI), *p* = 0.0112, Fig. 1D). Regarding the functional assessment of S1 sensory cortical region, we employed adhesive removal test and found that the IPC pre-treatment group showed a shortened removal time from the adhesive surface after the damaging ischemia surgery, compared with the group without IPC treatment over the experimental time period (Supplementary Fig. S1E). These results demonstrate that IPC confers significant ischemic tolerance phenotypes in motor and sensory cortical regions.

Next, we investigated whether IPC exerted the similar protective effects on the striatum against subsequent damaging ischemia. We analyzed the dorsal medial (DM), ventricular medial (VM), ventricular lateral (VL), and central amygdaloid nucleus (CeA) regions of the striatum (Supplementary Fig. S2A). Nissl- and NeuN-staining revealed that the 17 min-2VO caused significant loss of neurons in these regions (Supplementary Fig. S2B-C). IPC-pretreatment significantly reduced neuronal loss caused by the damaging ischemia in all tested regions in the striatum (DM: 703.2 ± 48.93 vs. 804.2 ± 47.80; *p* = 0.0090; CeA: 663.9 ± 40.22 vs. 816.2 ± 57.39, *p* = 0.0001; DL: 664.8 ± 27.65 vs. 788.9 ± 80.58, *p* = 0.0021; VM: 650.0 ± 38.09 vs. 780.6 ± 67.53, *p* = 0.0041l; VL: 697.7 ± 31.10 vs. 779.2 ± 46.64, *p* = 0.0061, Supplementary Fig. S2C). In addition, we assessed the correlated striatal functions of coordinated movement capacity of front paws by the Hanging wire test, and the correlated striatal motor and cognitive functions by the Open Field test [[28], [29], [30]]. As shown in Supplementary Fig. S2D-E, IPC+DI group significantly enhanced these performances that are correlated with striatal functions compared with the DI group (Open field test-72h: 68.02 ± 12.50 vs. 86.85 ± 7.606, *p* = 2.52×10^-5^, Supplementary Fig. S2E).

Similar to the cortex and striatum, we found that IPC also protected the neuronal integrity in the thalamus (Supplementary Fig. S3A-B). For the corresponding neurological test assessing the correlated thalamus functions, we measured the time interval between the first touch of a mouse on an adhesive tape and the time it took to completely remove the tape, which are correlated with the thalamus functions that are transmitted by sensation [26,27]. The result indicated that IPC+DI group exhibited significant alleviation of the correlated functional deficit of thalamus compared with the DI group at 24h, 48h and 72h after the damaging ischemic surgery (Supplementary Fig. S3C).

With respect to the hippocampus, we analyzed four Cornu Ammonis (CA) regions including the CA1- CA4 (Supplementary Fig. S4A) and dentate gyrus (DG) (Supplementary Fig. S5A) regions by Nissl staining. We found that 17 min-2VO caused injury to neurons in these regions (Supplementary Fig. S4B-C; Fig. S5B-C) as reported by others [[34], [35], [36]]. However, no prominent protective effect of IPC was observed in any of these hippocampal regions (DI vs. IPC+DI, Supplementary Fig. S4C, S5C). To confirm these observations, we performed NeuN staining to measure the neuronal loss in these regions. Consistently, we observed significant neuronal loss in the hippocampal DG, CA1 and CA2 regions. However, no significant rescue of neuronal loss by IPC could be noticed in these hippocampal regions (Supplementary Fig. S6). Moreover, ultrastructural observations from the transmission electron microscopy results indicate that IPC did not prominently mitigate the 17 min 2VO-induced injuries in the hippocampus, as evident by the swollen mitochondria with disintegrated cristae, large vacuole, and disintegrated endoplasmic reticulum in the IPC-pretreated hippocampal neurons (DI vs. IPC+DI, Supplementary Fig. S7).

To verify the ischemic tolerance conferred by IPC in these tested brain regions, we performed TUNEL and NeuN co-staining and showed that IPC alleviated the subsequent neuronal damages in the cortex (394.4 ± 50.95 (DI) vs. 144.7 ± 4.752 (IPC+DI), *p* = 2.97×10^-10^, Fig. 1E,F) and striatum (425.4 ± 82.31 (DI) vs. 217.3 ± 42.40 (IPC+DI), *p* = 0.0027, Supplementary Fig. S8) after 17 min-2VO. However, no significant effect of IPC on neuroprotection in the thalamus could be observed (Supplementary Fig. S3D).

### IPC alters SOD1 expression and nuclear accumulation in different brain regions

**Fig. 2:**
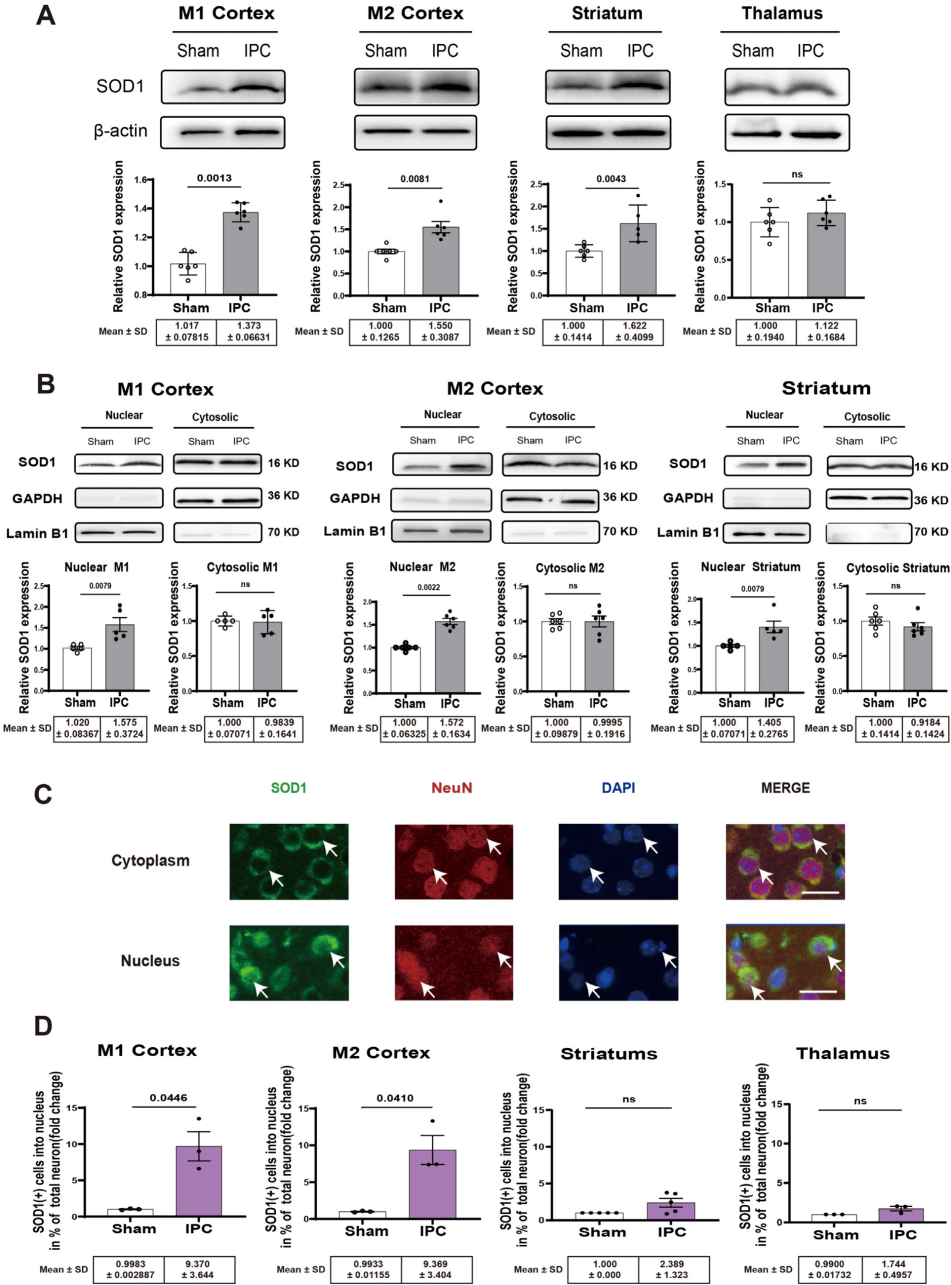
**IPC increases SOD1 nuclear localization in the cortical neurons. (A)** Expression of SOD1 protein in M1 cortex, M2 cortex, striatum and thalamus in sham and IPC treated mice. SOD1 expression level was determined by anti-SOD1 antibody. β-actin was used for the purpose of loading control. Error bars represent mean ± SD, n = 6 per group. *P* values (paired *t*-test) are indicated on the bars; ns, no significant difference. **(B)** The indicated brain regions were isolated and subjected to cell fractionation. The nuclear and cytoplasmic SOD1 levels were determined using anti-SOD1 antibody by Western blot. GAPDH and Lamin B1 were used as cytoplasmic and nuclear marker proteins, respectively. Error bars represent mean ± SD, n = 6 per group. *P* values (paired *t*-test) are indicated on the bars; ns, no significant difference. **(C)** Representative immunofluorescent images showing the cytoplasmic and nuclear localization of neuronal SOD1 in different brain regions. SOD1 was stained with a specific SOD1 antibody. Neuronal cells were recognized by NeuN antibody staining. Cell nuclei were visualized by DAPI staining. Arrows indicate the cells showing prominent cytoplasmic SOD1 (upper panel) and nuclear SOD1 (lower panel), respectively. Scale bars = 20 μm. **(D)** Quantification showing that changes of nuclear SOD1 neurons in different brain regions in sham and IPC-treated mice. Error bars represent mean ± SD (n = 3–5). *P* values (Wilcoxon matched-pairs signed rank test) are indicated on the bars; ns, no significant difference.

After identifying that the cortex and striatum were the IPC-sensitive brain regions, we investigated the possible involvement of SOD1 in mediating the protective mechanism conferred by IPC. First, we determined the SOD1 expression levels in these brain tissues after IPC stimulus in comparison with the corresponding tissues in sham surgery control. By Western blot analysis, we observed that the expression of SOD1 was significantly increased in the M1 (1.017 ± 0.078 vs. 1.373 ± 0.066, *p* = 0.0013) and M2 cortex (1.00 ± 0.1265 vs. 1.550 ± 0.3087, *p* = 0.0081) and striatum tissues (1.00 ± 0.1414 vs. 1.622 ± 0.4099, *p* = 0.0043) collected at 72h after IPC treatment by 6 min-2VO compared with sham control (Fig. 2A), whereas we did not notice any significant alternation of SOD1 expression in the thalamus tissue (Fig. 2A). These results indicate that SOD1 expression is correlated with the transient global IPC-induced ischemic tolerance.

We have previously shown that SOD1 accumulates in the nucleus after environmental stressful conditions in budding yeast and human fibroblast [37]. Our observation that ischemic preconditioning stimulus increased SOD1 expression in the cortex and striatum prompted us to ask whether IPC could alter the nuclear localization of SOD1 in these brain regions. We performed the cell fractionation experiments from these brain regions to separate the nuclear and cytoplasmic fractions, as indicated by the corresponding marker proteins Lamin B1 and GAPDH, respectively. Our results showed that SOD1 was significantly increased in the nuclear fraction after IPC treatment in the cortex (M1: 1.020 ± 0.08367 vs. 1.575 ± 0.3724, *p* = 0.0079; M2: 1.000 ± 0.06325 vs. 1.572 ± 0.1634, *p* = 0.0022, Fig. 2B) and striatum (1.000 ± 0.07071 vs. 1.405 ± 0.2765, *p* = 0.0079, Fig. 2B). In addition, we further used NeuN and SOD1 double labeling to visualize the SOD1 cytoplasmic and nuclear localization (Fig. 2C) specifically in neurons in the tested brain regions. Quantification of neurons showing nuclear and cytoplasmic SOD1 in different brain regions revealed that IPC treatment most prominently increased neuronal SOD1 nuclear accumulation in the M1 and M2 cortex among the tested brain regions (Fig. 2D). These results suggest that SOD1, particularly in cortical neurons, mediates ischemic tolerance where significant increase in SOD1 expression and nuclear accumulation take place in the neurons of cortex after IPC stimulus.

### SOD1 is required for IPC-induced ischemic tolerance in cortical neurons

**Fig. 3:**
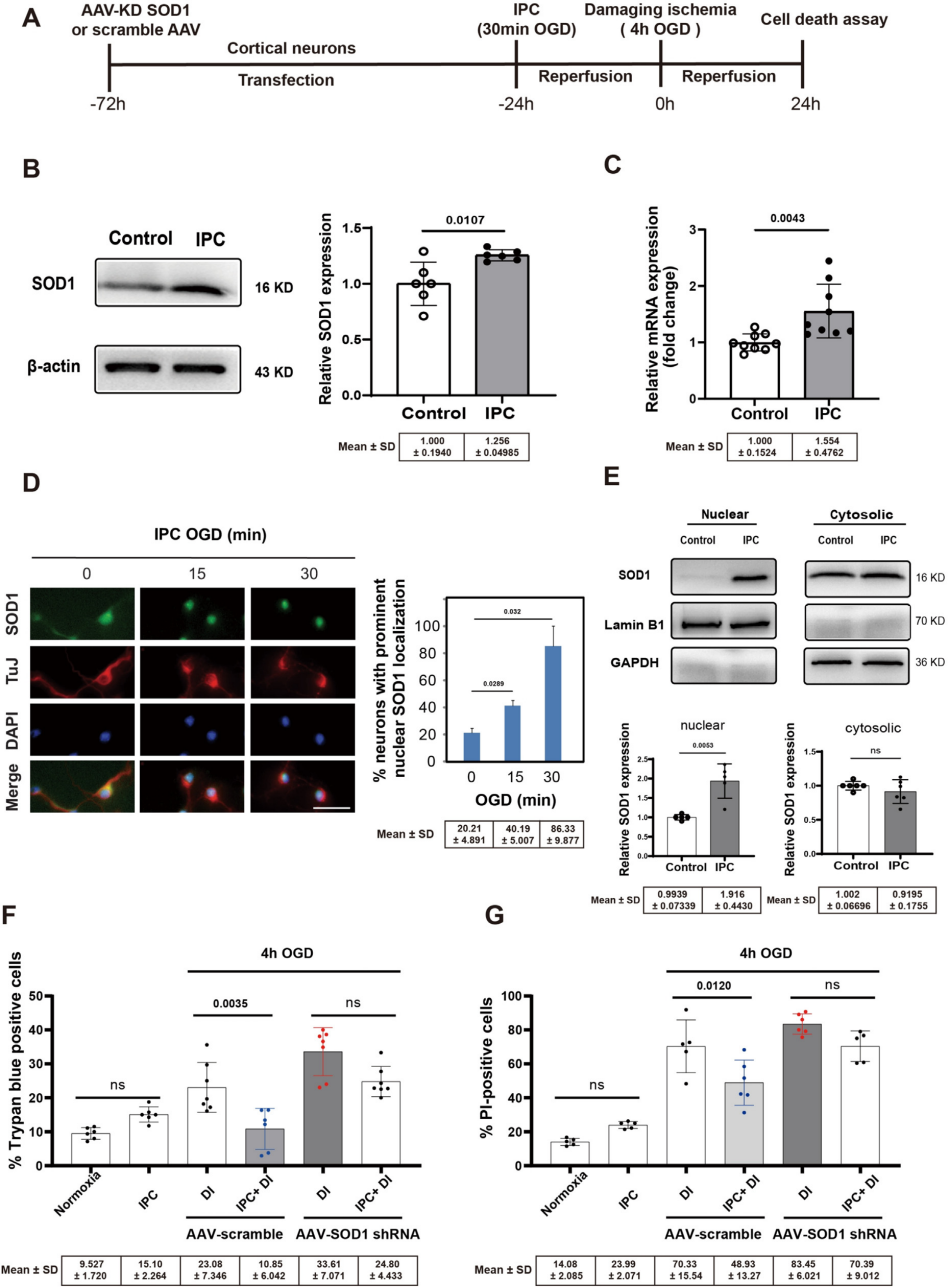
**SOD1 plays a critical role in ischemic preconditioning-induced tolerance in cortical neurons. (A)** Experimental scheme for the *in vitro* model of IPC-induced ischemic tolerance in cultured cortical neurons. **(B)** SOD1 protein expression level in the cultured cortical neurons treated with or without IPC for 24 h. SOD1 protein levels were determined by Western blot using anti-SOD1 antibody. Beta-actin was used as a loading control. Error bars represent mean ± SD (n = 6). *P* value (paired *t*-test) is indicated on the bars. **(C)** SOD1 mRNA expression level in cultured cortical neurons treated with or without IPC for 24 h was determined by RT-qPCR. Error bars represent mean ± SD (n = 9). *P* value (paired *t*-test) is indicated above the horizonal line comparing the paired bars. **(D)** Immunofluorescence staining of SOD1 in the cultured cortical neurons at the indicated time points after IPC treatment. SOD1 was detected by anti-SOD1 antibody. TuJ and DAPI were used to stain neurons and nucleus, respectively. Scale bar = 50 μm. Right panel indicates the quantification results of % neurons with nuclear SOD1 localization. Error bars indicate mean ± SD, n = 6 per group. *P* values (one-way ANOVA with Tukey’s multiple comparison test) are indicated on the bars. **(E)** Nuclear and cytoplasmic SOD1 protein levels were determined by cell fractionation experiment with Western blot analysis in cultured cortical neurons after IPC treatment for 24 h. LAMIN B1 and GAPDH were respectively used for nuclear and cytoplasmic markers. Lower panel indicates the quantification results. Error bars represent mean ± SD (n = 6). *P* value (paired *t*-test) is indicated on the bar; ns, no significant difference. **(F)***In vitro* IPC-induced ischemic tolerance model in cultured cortical neurons transduced with AAV-SOD1 shRNA or AAV-scramble control as depicted in panel A. Neuronal cell viability was measured by Trypan Blue staining method. Error bars represent mean ± SD (n = 6). *P* value (one-way ANOVA with Tukey’s multiple comparison test) is indicated above the horizonal line comparing the paired bars; ns, no significant difference. **(G)** Same experimental procedure as (F) but neuronal cell viability was measured by propidium iodide (PI) staining method. Error bars represent mean ± SD (n = 5). *P* value (one-way ANOVA with Tukey’s multiple comparison test) is indicated above the bar; ns, no significant difference.

According to the above results, we next focused on the mechanistic role of SOD1 in the cortical neurons for establishing ischemic tolerance. We used cultured cortical neurons and the well-established *in vitro* oxygen-glucose deprivation (OGD)-induced ischemic tolerance cell-based model as depicted in Fig. 3A [17,38,39]. First, we confirmed that IPC by a brief OGD treatment for 30 min increased expression of SOD1 in the cortical neurons in both protein level and mRNA level as determined by Western blot (Fig. 3B) and RT-qPCR (1.000 ± 0.1524 vs. 1.554 ± 0.4762, *p* = 0.0043, Fig. 3C), respectively. Then, we performed immunofluorescent staining in the cultured cortical neurons. Notably, IPC caused rapid accumulation of SOD1 in the nucleus (Fig. 3D). Result of cell fractionation analysis also verified that SOD1 significantly accumulated in the nuclear fraction in the cortical neurons after IPC treatment (0.9939 ± 0.07339 vs. 1.916 ± 0.4430, *p* = 0.0053, Fig. 3E).

To determine whether SOD1 played a role in the IPC-induced ischemic tolerance, we knocked down SOD1 by transducing cortical neurons with virus-expressing shRNA against SOD1 (Supplementary Fig. S9). Then, we performed the IPC-induced ischemic tolerance in the *in vitro* model and measured the cell viability in cortical neurons in scramble and SOD1-knockdown groups. Consistent with previous report [17,38,39], we showed that the brief IPC stimulus by the OGD treatment did not significantly change the cell viability, and that damaging ischemia by 4h OGD caused significant cell death (Fig. 3F). We confirmed that IPC significantly enhanced viability of cortical neurons against the subsequent damaging ischemia as determined by Trypan blue staining (Fig. 3F). However, SOD1 knockdown significantly abolished the IPC-induced protective effect (Scramble: 23.08 ± 7.346 vs. 10.85 ± 6.042, *p* = 0.0035; SOD1 knockdown: 33.61 ± 7.071 vs. 24.80 ± 4.433, *p* > 0.05). Essentially, similar results were observed by using PI-staining method for measurement of neuronal cell viability (Fig. 3G).

### SOD1 regulates transcriptional activity of CXCR4 in response to ischemic preconditioning in cortical neurons

**Fig. 4:**
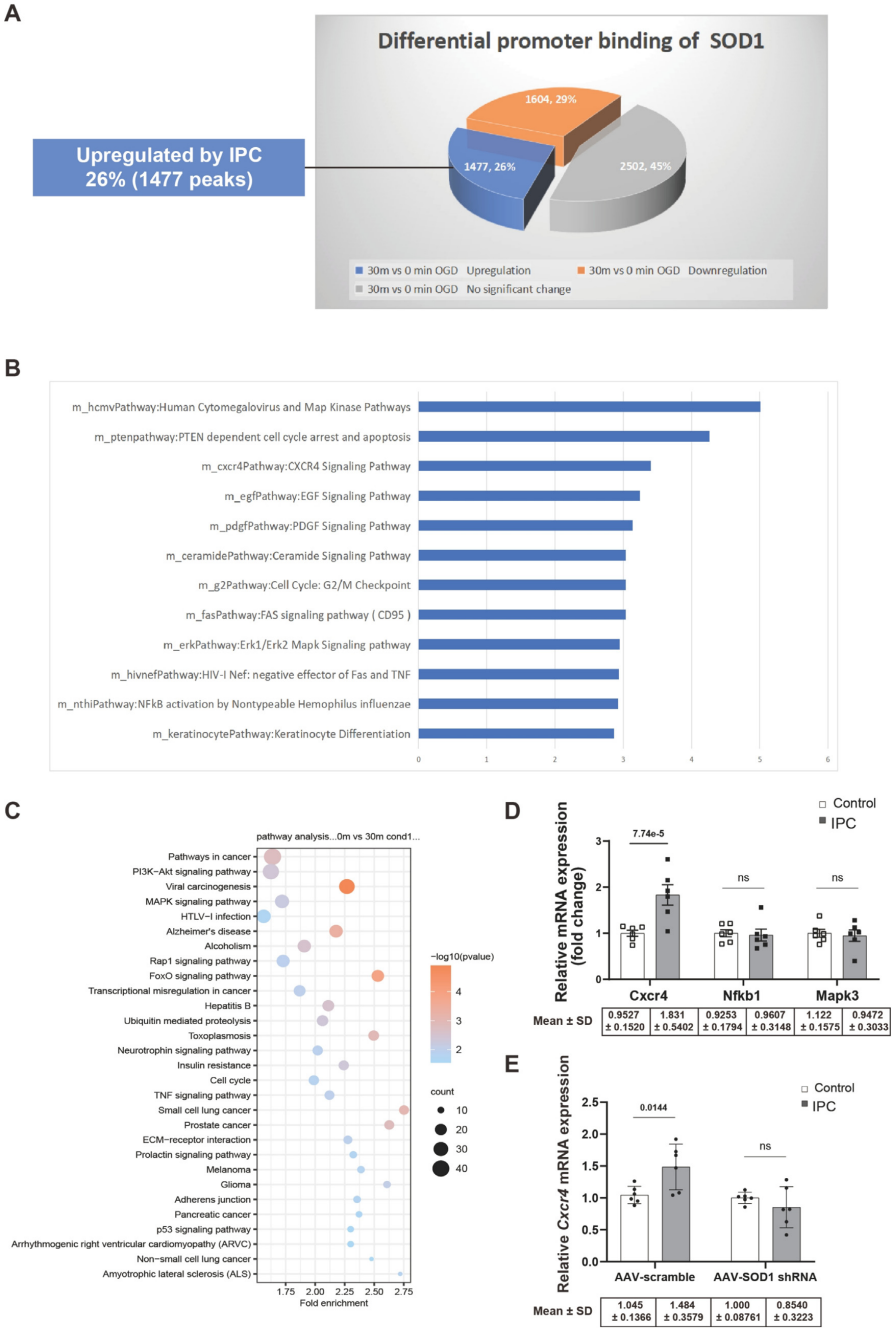
**Change of SOD1-binding peaks at promoters after 30 min OGD preconditioning stimulus. (A)** Pie chart showing the numbers of 30 min OGD-induced up-regulation of SOD1-binding peaks, down-regulated SOD1-binding peaks and the peaks that did not show any significant changes, compared with 0 min OGD-control cells. **(B)** Specific gene set enriched pathways as revealed by the Database for Annotation, Visualization, and Integrated Discovery (DAVID). Results indicate the pathways enriched by the genes showing up-regulated SOD1-binding peaks at the promoter in the 30 min OGD preconditioned cells vs 0 min OGD control cells. **(C)** BioCarter pathway analysis indicates the key pathways enriched by genes showing differentially up-regulated SOD1-binding at the promoter regions in the 30 min OGD-preconditioned cells vs 0 min OGD control cells. **(D)** Expression of the indicated SOD1-target genes in CXCR4 pathway was determined by RT-qPCR in cultured cortical neurons treated with or without 30 min IPC and 24 h reperfusion. Error bars represent mean ± SD (n = 6). *P* value (paired *t*-test) is indicated on the bar; ns, no significant difference. **(E)** Cultured cortical neurons were infected with AAV-scramble or AAV-SOD1 shRNA before the *in vitro* IPC-induced ischemic tolerance procedure as described in 6A. Expression of CXCR4 was determined as described in (A). Error bars represent mean ± SD (n = 6). *P* values (paired *t*-test) are indicated above on the bar; ns, no significant difference.

Accumulating lines of evidence from us and others have revealed that SOD1 is an evolutionarily conserved chromatin-binding protein under different pathophysiological conditions [37,[40], [41], [42]]. Given that SOD1 was rapidly translocated to the nucleus of cortical neurons in response to IPC treatment in our *in vitro* (Fig. 3D) and *in vivo* results (Fig. 2C-D), we asked whether the nuclear SOD1 plays a critical role in transcriptional regulation upon IPC stimulus. To this end, we treated the cultured cortical neurons in normoxic or ischemic OGD medium for 30 min to induce IPC, and then performed CUT&Tag-sequencing analysis to map the DNA binding profile of SOD1, because this is a sensitive method to detect the *in vivo* chromatin binding activity of protein factors [17,42,43]. As shown in Fig. 4A, our CUT&Tag-sequencing result revealed that IPC-treated cells significantly altered the SOD1-promoter binding pattern compared with the control cells. Intriguingly, there were 1,477 upregulated SOD1-binding peaks, and 1,604 downregulated SOD1-binding peaks at the promoter regions (Fig. 4A). Integrative analyses of the upregulated SOD1-promoter binding genes by DAVID and BioCarter pathway analyses further revealed CXCR4 pathway (*Cxcr4*, *Nfkb1*, *Mapk3*), ubiquitin signaling pathway (*Btbd11, Mylip, Rnf145*), redox signaling pathway (*Glrx2, Tmx4, Prdx3*), Foxo signaling pathway (*Atm, Foxo3, Fbxo7*), Mapk signaling pathway (*Nfkb1, Mapk14, Mapk3*), oxidative stress-induced signaling pathway (*Mapk14, Mdm2, Mapk3*), and histone signaling pathway (*Atf2, Kat8, Noca2*) were among the top enriched pathways with functional relevance activated by IPC treatment in cortical neurons (Fig. 4B-C). To investigate whether the observed upregulation of SOD1-binding to these gene promoters was correlated to the transcriptional activation of these genes, we performed RT-qPCR in the cultured cortical neurons, and showed that CXCR4 expression was significantly enhanced after IPC treatment (0.9527 ± 0.1520 vs. 1.831 ± 0.5402, *p* = 7.74×10^-5^, Fig. 4D). To further confirmed whether other genes in the CXCR4 pathway were upregulated, we screened the known components in the CXCR4 pathway and identified that the expression levels of multiple components of the CXCR4 pathway were altered after IPC treatment (Supplementary Fig. S10A). With respect to other pathways, however, the upregulated SOD1-promoter binding genes in these pathways were not prominently associated with their transcriptional activation after IPC treatment in the cultured cortical neurons (Supplementary Fig. S10B, E-H). These results suggest that the CXCR4 pathway is involved in SOD1-mediated transcriptional activation in response to IPC in cultured cortical neurons. To verify this observation *in vivo*, we performed IPC by 6 min-2VO surgery in the mouse model and collected M1 and M2 cortical tissues. Consistently, RT-qPCR results showed that both M1 and M2 cortical tissues exhibited significant increased expression of CXCR4 and the other components in this pathway (Supplementary Fig. S10C-D).

To determine whether SOD1 was required for the observed upregulated of CXCR4 expression in response to IPC treatment in cortical neurons, we knocked down SOD1 in cortical neurons by AAV-shRNA, and compared the IPC-induced transcriptional activity of CXCR4 with neurons transduced with scramble control. As shown in Fig. 4E, SOD1 knockdown completely abolished the IPC-induced upregulation of CXCR4 expression (scramble: 1.045 ± 0.1366 vs. 1.484 ± 0.3579, *p* = 0.0144; SOD1 knockdown: 1.000 ± 0.08761 vs. 0.8540 ± 0.3223, *p* > 0.05).

### IPC-induced SOD1-mediated upregulation of CXCR4-STAT3 signaling pathway in cortical neurons alleviates apoptosis caused by damaging ischemia

**Fig. 5:**
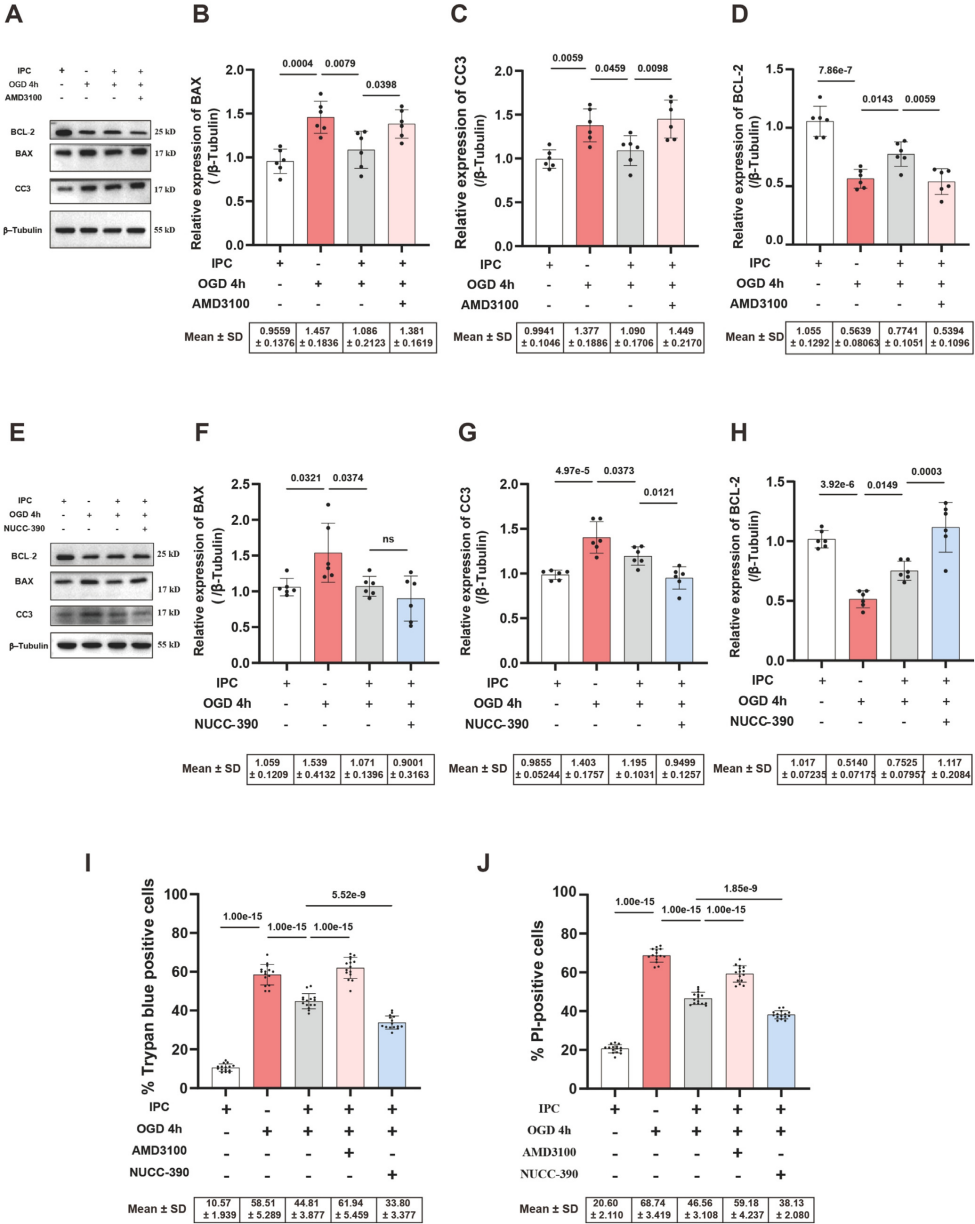
**C**XCR**4 regulates apoptosis in IPC-induced ischemic tolerance in cultured cortical neurons. (A)** Western blotting was used to determine the expression levels of of apoptotic proteins BAX, cleaved caspase 3 (CC3) and BCL2 in cultured cortical neurons in the *in vitro* IPC-induced ischemic tolerance model with or without the treatment of the CXCR4 inhibitor AMD3100. β-Tubulin serves as a loading control. **(B-D)** Quantification result of the expression level of BAX (B), CC3 (C), and (D) BCL-2. Error bars indicate mean ± SD, n = 6. **(E)** Western blot analysis of the apoptotic proteins in cultured cortical neurons in the *in vitro* IPC-induced ischemic tolerance model in the absence or presence of the CXCR4 activator NUCC-390. **(F-H)** Quantification of the expression level of BAX (F), CC3 (G), and (H) BCL-2 in panel E. Error bars represent mean ± SD (n = 6). **(I-J)** Cell viability assays of the cultured cortical neurons in the *in vitro* IPC-induced ischemic tolerance model in the absence or presence of AMD3100 or NUCC-390. Neuronal cell viability was measured by trypan blue staining method (I) or propidium iodide (PI) staining method (J). Error bars indicate mean ± SD (n = 15). Statistical analysis in this figure was performed using one-way ANOVA with Tukey's multiple comparisons test. *P* values are indicated on the bar; ns, no significant difference.

CXCR4 is known to be commonly expressed in the immune cells and neural precursor cells for regulation of neuroinflammation and neurogenesis, respectively [44]. However, its role in cortical neurons remains largely unexplored. Our observations that CXCR4 was upregulated in response to IPC (Fig. 4D) which was associated with reduced neuronal cell death upon the subsequent damaging ischemia (Fig. 3F-G) prompted us to test whether CXCR4 plays a role in regulating apoptosis in cortical neurons in the IPC-induced ischemic tolerance. Using the *in vitro* model, we pretreated cortical neurons with AMD3100 (the specific CXCR4 inhibitor) and performed IPC-induced ischemic tolerance experiment. As shown in Fig. 5A-C, the pro-apoptotic marker proteins BAX and cleaved caspase 3 (CC3) were increased in their expression after 4h OGD-induced damaging ischemia treatment (Fig. 5B,C, second column) compared to IPC-only group (Fig. 5B,C, first column), and IPC+4h OGD group showed significantly alleviated expression of BAX (1.457 ± 0.1836 vs. 1.086 ± 0.2123, *p* = 0.0079, Fig. 5B) and CC3 (1.377 ± 0.1886 vs. 1.090 ± 0.1706, *p* = 0.0459, Fig. 5C). Notably, inhibition of CXCR4 by AMD3100 treatment abolished the protective effect against apoptosis conferred by IPC (BAX: 1.086 ± 0.2123 vs. 1.381 ± 0.1619, *p* = 0.0398; CC3: 1.090 ± 0.1706 vs. 1.449 ± 0.2170, *p* = 0.0098, Fig. 5B,C, 4th column). Consistently, we observed the opposite trends in the expression of anti-apoptosis marker protein BCL2 (Fig. 5A, D). These results reveal that CXCR4 is required for the IPC-induced ischemic tolerance through alleviation of apoptosis in cortical neurons.

To further demonstrate the positive role of CXCR4 in the observed IPC-induced protection against apoptosis caused by subsequent damaging ischemia, we pretreated cortical neurons with the CXCR4 activator NUCC-390 before the IPC-induced ischemic tolerance. Compared with the IPC+4h OGD group, CXCR4-activated group further reduced the pro-apoptotic marker protein CC3 expression (1.195 ± 0.1031 (3rd column) vs. 0.9499 ± 0.1257 (4th column), *p* = 0.0121, Fig. 5E-G), with enhanced expression level of the anti-apoptotic marker protein BCL2 (0.7525 ± 0.0796 (3rd column) vs. 1.117 ± 0.2084 (4th column), *p* = 0.0003, Fig. 5H). Moreover, we confirmed that the CXCR4-mediated apoptotic marker protein expressions were correlated with cell viability in the cortical neuron (Fig. 5I,J). Taken together, these results illustrate that CXCR4 promotes cortical neuronal cell viability by anti-apoptotic mechanism in the IPC-induced ischemic tolerance response in cortical neurons.

**Fig. 6:**
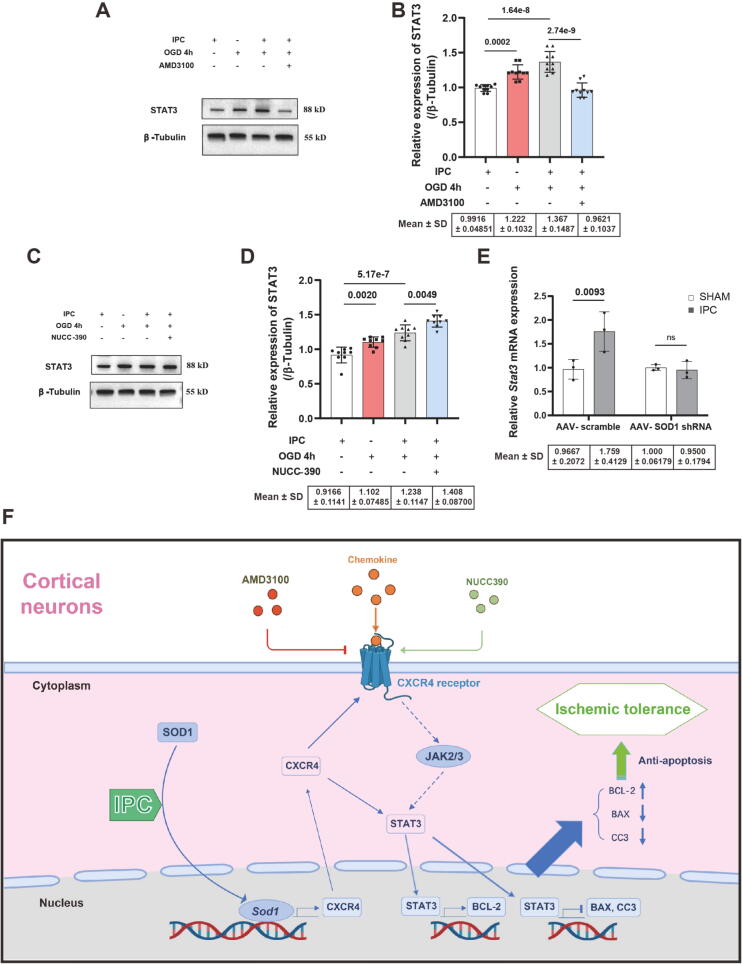
**SOD1-CXCR4-STAT3 axis regulates ischemic tolerance in cortical neurons. (A)** Western blot analysis of the protein expression level of STAT3 in the cortical neurons of the *in vitro* ischemic tolerance model with or without the treatment of the CXCR4 inhibitor AMD3100. STAT3 protein expression level was determined by specific STAT3 antibody, and β-Tubulin served as a loading control. **(B)** Quantification of results in (A). Error bars represent mean ± SD (n = 10). *P* values were determined by one-way ANOVA with Tukey’s multiple comparison test. **(C)** STAT3 protein expression determination by Western blot analysis in cortical neurons in the *in vitro* ischemic tolerance model. Cells were treated with or without the CXCR4 activator NUCC-390. **(D)** Quantification of results in (C). Error bars represent mean ± SD (n = 9). *P* values were determined by one-way ANOVA with Tukey’s multiple comparison test. **(E)** Cultured cortical neurons were infected with AAV-scramble or AAV-SOD1 shRNA before the *in vitro* IPC-induced ischemic tolerance procedure as described in 6A. Expression level of STAT3 was determine by RT-qPCR. Error bars represent mean ± SD (n = 3). Statistical analysis was performed using Wilcoxon matched-pairs signed rank test; *p* value is indicated on the bar; ns, no significant difference. **(F)** A working model of this study. See text for detailed description. ↓ indicates activation; ⊥ indicates inhibition; dotted arrow indicates hypothetical link. IPC, ischemic preconditioning; CC3, cleaved caspase 3.

Compelling evidence has revealed that STAT3 promotes survival in a number of cell types under stressful conditions [45]. In addition, it has been reported that CXCR4 regulates apoptosis via promoting STAT3-mediated apoptosis in cancer cells [46]. To determine whether CXCR4 in cortical neurons plays a role in STAT3 expression, we used the *in vitro* IPC-induced ischemic tolerance model and determined the change of STAT3 expression level by modulation of CXCR4 activity in cortical neurons. As shown in Fig. 6A-B, the IPC-induced ischemic tolerance was accompanied by increased expression of STAT3. Notably, CXCR4 inhibition resulted in significant downregulation of STAT3 expression (1.367 ± 0.1487 (3rd column) vs. 0.9621 ± 0.1037 (4th column), *p* = 2.74×10^-9^, Fig. 6A,B), whereas CXCR4 activation by NUCC-390 treatment exhibited an opposite effect (1.238 ± 0.1147 (3rd column) vs. 1.408 ± 0.087 (4th column), *p* = 0.0049, Fig. 6C,D). Finally, we confirmed whether SOD1 is required for the IPC-induced upregulation of STAT3 expression in cortical neurons. We showed that knockdown of SOD1 abolished the IPC-induced upregulation of STAT3 (1.000 ± 0.06179 (3rd column) vs. 0.95 ± 0.1794 (4th column), *p* > 0.05, Fig. 6E). Collectively, these results suggest that SOD1-CXCR4-STAT3 pathway regulates apoptosis in cortical neurons for establishing the IPC-induced ischemic tolerance.

## Discussion and conclusion

In this study, we demonstrate that ischemic preconditioning (IPC)-induced tolerance occurs in different brain regions including cortex, striatum and thalamus, but unexpectedly not in the hippocampus in the mouse model of ischemic tolerance. Additionally, we discover that SOD1 plays a critical role specifically in the cortical neurons for establishment of ischemic tolerance. Mechanistically, we demonstrate that ischemic preconditioning induces rapid SOD1 nuclear translocation. Upon IPC stimulus, SOD1 enhances its binding to gene promoter in cortical neurons for upregulation of CXCR4 transcriptional regulation. In addition, we establish that STAT3 is the downstream effector of the resultant activated CXCR4 pathway which is required for the suppression of apoptosis via upregulation of anti-apoptotic and downregulation of pro-apoptotic proteins in cortical neurons in response to IPC stimulus. We also identified that treatment of CXCR4 agonist such as NUCC-390 could significantly alleviate the subsequent ischemic damage (Fig. 6F). These results demonstrate the potential of CXCR4 as a novel target for inducing ischemic tolerance and offer insight into the therapeutic strategy for treatment of stroke.

SOD1 has been linked to diverse diseases, including amyotrophic lateral sclerosis (ALS), premature aging and age-associated pathologies such as macular degeneration and tumorigenesis [47]. SOD1 is generally considered as a cytoplasmic protein with its major role as antioxidant. However, emerging evidence has shown that SOD1 can also be exist in the nucleus under various pathophysiological conditions [48]. Intriguingly, the nuclear SOD1 can exhibit non-canonical functions in addition to its superoxide dismutation catalytic activity [48]. By using the recently developed protein-chromatin profiling technique with high resolution and sensitivity, we discover that SOD1 binds to the promoter of CXCR4 which is associated with its transcriptional activation. In agreement with the current study, Barbosa *et al*. has also reported that SOD1 has a strong binding affinity with DNA in nerve cells [40]. In addition, by systematically analyzing the ability of various human recombinant proteins to DNA binding affinity, it has been found that human SOD1 can also bind to DNA [41]. Our previous study in the budding yeast found that oxidative stress induces SOD1 nuclear accumulation and chromatin binding to stress-responsive genes [37]. Subsequent study by Li *et al*. further showed that mammalian SOD1 binds to chromatin in HeLa cells. Using chromatin immunoprecipitation-sequencing analysis, they demonstrate that SOD1 binds to ALS-and tumor-associated genes [42]. Moreover, SOD1 is involved in downregulation of *PTK6* (the tumor suppressor gene) and upregulation of several different oncogenes, such as *FGFR4*, *TUSC2*, and *CRTC3* [42]. These studies reveal that SOD1 may regulate transcriptional functions for cell growth and survival. Therefore, these findings demonstrate the DNA binding activity of SOD1 is evolutionarily conserved, and suggest that SOD1 plays a more intimate functional role in regulation of transcription in the nucleus in different cell types. Although we only focused on neuronal CXCR4 pathway in this study, the possibility that SOD1 may also regulate other IPC-responsive genes involved in ischemic tolerance cannot be ruled out. Future study is required to explore the additional SOD1-target genes and their collaborative functions for establishment of ischemic tolerance in neurons as well as in other brain cell types.

CXCR4 is known as a member of the chemokine family that belongs to a G-protein-coupled receptor expressed mainly in hematopoietic and immune system. In addition to its well-known function for regulation of cell migration, CXCR4 regulates different cellular functions, including differentiation, growth, survival, and apoptosis [49,50]. In this study, we demonstrate that CXCR4 is the SOD1-target gene which modulates IPC-induced ischemic tolerance in cortical neurons. Consistent with our finding, it has also been reported that CXCR4 is expressed in neurons in the cerebral cortex [51,52]. Interestingly, treatment of CXCR4 agonist has been shown to strongly promote regeneration of degenerated motor axonal terminals [53]. Notably, a study has shown that the expression of CXCR4 is reduced in SOD1 mutant mouse species [54]. These results suggest that CXCR4 is a major SOD1 target gene in different cell types and pathophysiological conditions. It would be interesting to future explore the other potential intracellular functions of SOD1-mediated CXCR4 expression.

Notably, a recent study using the conditional monocyte-specific CXCR4 knockout approach has reported that monocyte-derived macrophages in the *Cxcr4* conditional knockout brain show reduced expression of several genes that govern the type-I-interferon-activated state, damage-associated molecular pattern recognition, and the responses to cytokine and interferon [55]. Given that these genes are required for improved outcome in the stroke model [56], these results suggest that CXCR4 participates in promoting the protective immunological defense response. Thus, we speculate that CXCR4 agonist may also promote the protective immunological defense activity after stroke, in addition to its direct role in enhancing protection in neurons against apoptosis as observed in this study. Future works will be important to verify this hypothesis.

In the current study, we demonstrate that STAT3 is a downstream effector of CXCR4 pathway in cortical neurons responsible for suppression of apoptosis and the acquired IPC-induced ischemic tolerance. In accordance with our finding, Groopman and co-workers have reported that the CXCR4 pathway can activate Janus kinases/signal transducers and activators of transcription (JAK/STAT) in hematopoietic progenitor cells [57]. An *in vitro* study using the non-small cell lung cancer cell line also demonstrates CXCR4 activation causes the reduction of cisplatin-mediated cell apoptosis through JAK2/STAT3 signaling pathway [46]. It remains unknown whether in cortical neurons, activation of CXCR4 could upregulate STAT3 via JAK or other signaling mechanism. Consistent with our finding, BCL-2 and BAX have been shown to be the targets of STAT3 because the inhibition of BAX has been shown to promote cell survival after STAT3 activation [58]. In addition, 3-formylchromone-mediated downregulation of STAT3 could inhibit the expression of BCL-2 and BCL-XL, suggesting that 3-formylchromone suppresses the STAT3 pathway and thus promoting apoptosis in cancer cells [58,59]. Furthermore, Balic *et al.*, reported that the treatment of metastatic cancer stem cells with chloroquine can significantly diminish their viability through the inhibitory effect on CXCR4 signaling pathway, leading to inactivation of ERK and STAT3, resulting in reduction of cell viability [60]. In cardiac myocytes, JAK2-STAT signaling activation could mitigate the extent of myocardial injury [61]. Kim *et al.* reported in A549 and H460 cell lines that CXCR4 overexpression could improve clonogenic survival, and CXCR4 expression is correlated with STAT3 activation. They further showed that the inhibition of STAT3 leads to the enhanced breast tumor cell apoptosis by down-regulating the expression of BCL-XL and BCL-2 [46]. Interestingly, a study using toxoplasma-induced brain injury showed that increased mRNA expression of the CXCR4 agonist CXCL12, and the elevation of STAT3 expression could regulate protective innate immunity and T cell-mediated protective immunological responses against cerebral infection with *Toxoplasma gondii* [62]. Studies also pointed out the possibility of feed-back regulation of STAT3 to CXCR4 activity via expression of its agonist [63]. Therefore, the detailed mechanism by which SOD1-CXCR4-STAT3 axis orchestrates ischemic tolerance in cortical neurons warrants further investigation in the future. While the roles of neuroinflammatory and immune responses such as cytokines, TLR signaling and Th1/Th2 activities in post-ischemic inflammation are well-established [10], our current study provides a novel perspective on the specific and dynamic role of SOD1 in modulating neuroinflammation and neuronal survival for future in-depth investigations not only in ischemic but also in other neurological pathologies.

The limitations of this study include only focusing on the CXCR4 pathway and not yet exploring the other less prominent SOD1-regulated genes that might also mediate the neuronal ischemic tolerance phenotype. Moreover, whether JAK family members are involved in mediating CXCR4-STAT3 signal remained to be elucidated. In addition, the potential effect of CXCR4 agonist on immunological and inflammatory responses in the brain tissue after IPC and injurious ischemia has not yet been fully understood. Regarding methodological limitation, we only used TEM for ultrastructural analysis of neuronal organelles, future study using immuno-TEM would further provide insight into the SOD1 functions. Additionally, we only used the bulk tissue samples for analyses, future study using the single cell-multi-omic technologies would extend the roles of SOD1 and CXCR4 in other brain cell types.

## Conclusion

Our findings in this study indicate that CXCR4 is a vital target and that CXCR4 agonists such as NUCC-390 may have the potential to be developed as a prophylactic agent for individuals who are anticipated to experience a high risk of ischemic injuries in the central nervous system and other peripheral organs. For example, patients who are going to undergo the carotid endarterectomy surgery, aneurysm-associated clipping of blood vessel, arterial tumor dissection procedure, vascular stenting surgery, coronary bypass surgery, cardiac value replacement or carotid stenosis surgery may have a very high risk of suffering from injurious ischemia during these surgeries. The CXCR4 agonists may therefore serve as prophylactic agents to boost the ischemic tolerance capability of these patients right before the execution of these surgeries. Understanding the ischemic tolerance mechanism mediated by SOD1 revealed by this study may also provide novel insight into the viable strategies for treatment of ischemic stroke and other ischemic pathologies.

## Author contributions

Chi Kwan Tsang and Anding Xu conceived the project. Ming-Xi Li, Guangpu Su and Ying Zhou planned, designed and executed experiments. Chi Kwan Tsang, Ming-Xi Li, Guangpu Su and Zhenguo Yang analyzed the data and produced figures. Chi Kwan Tsang, Ming-Xi Li and Guangpu Su wrote the manuscript. All authors contributed to the discussion of the results.

## Fundings

This work was supported by the Guangdong Basic and Applied Basic Research Foundation, China (2023B1515120035, 2024A1515012035), National Natural Science Foundation of China (No. 82471313, 81974210), and the Science and Technology Planning Project of Guangdong Province, China (No. 2020A0505100045).

## Declaration of competing interest

The authors declare that they have no known competing financial interests or personal relationships that could have appeared to influence the work reported in this paper.
