# Supplementary material for: SOD1 regulates CXCR4 transcription in cortical neurons for establishment of cerebral ischemic tolerance
Source: J Adv Res. 2025 Oct 23;85:899–917. doi: 10.1016/j.jare.2025.10.039 (PMC13316499; doi:10.1016/j.jare.2025.10.039)
Supplement: Supplementary Data 1 [file mmc1.docx]

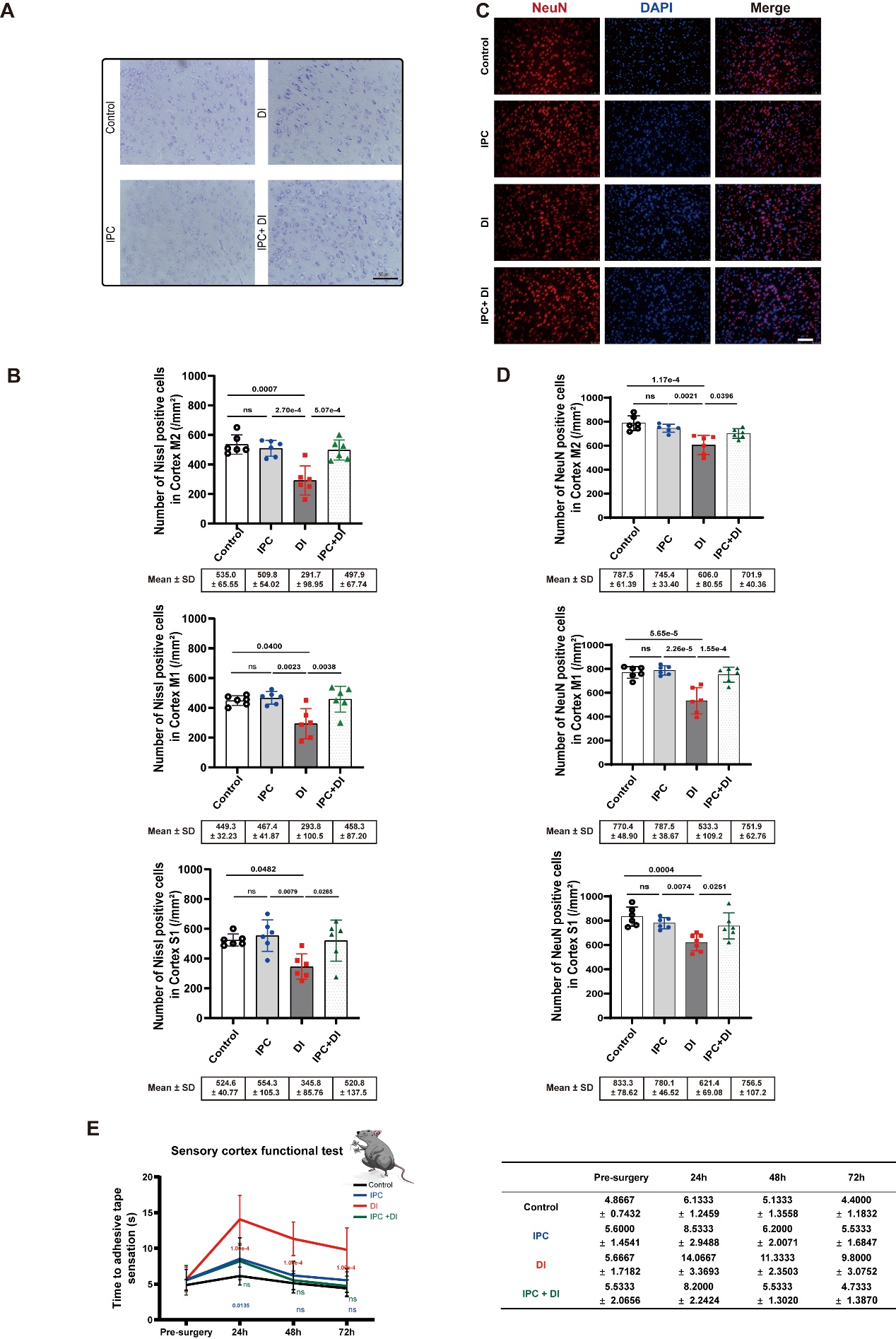


**Supplementary Figure S1. IPC-induced ischemic tolerance in the cerebral cortex. (A)** Representative images showing the neuronal injury measured by Nissl staining in cortical region 3 days after the 17 min 2VO surgery. Scale bar = 50 μm. **(B)** Quantification of result in (A). Error bars indicate mean ± SD, n = 6 per group. Statistical analysis was performed using one-way analysis of variance (ANOVA) coupled with Tukey's multiple comparisons test. *P* values are indicated on top of the bars; ns, no significant difference. **(C)** Representative images of Neuron density in different cortical regions by NeuN-Immunofluorescence staining. Scale bar = 100 μm. **(D)** Quantification of result in (C). Error bars indicate mean ± SD, n = 6 per group. Statistical analysis was performed using one-way analysis of variance (ANOVA) coupled with Tukey's multiple comparisons test. *P* values are indicated on top of the bars; no significant difference. **(E)** Sensory cortical function was measured by the correlated Adhesive removal test which recorded the time for the sticky sensation of the adhesive tap at 24h, 48h, and 72h after the 17 min 2VO surgery. Error bars indicate mean ± SD, n = 8 per group. Statistical analysis was performed using two-way ANOVA coupled with Dunnett's multiple comparisons. *P* values for DI vs. control and IPC + DI vs. DI are indicated by blue and red numbers, respectively; ns, no significant difference.


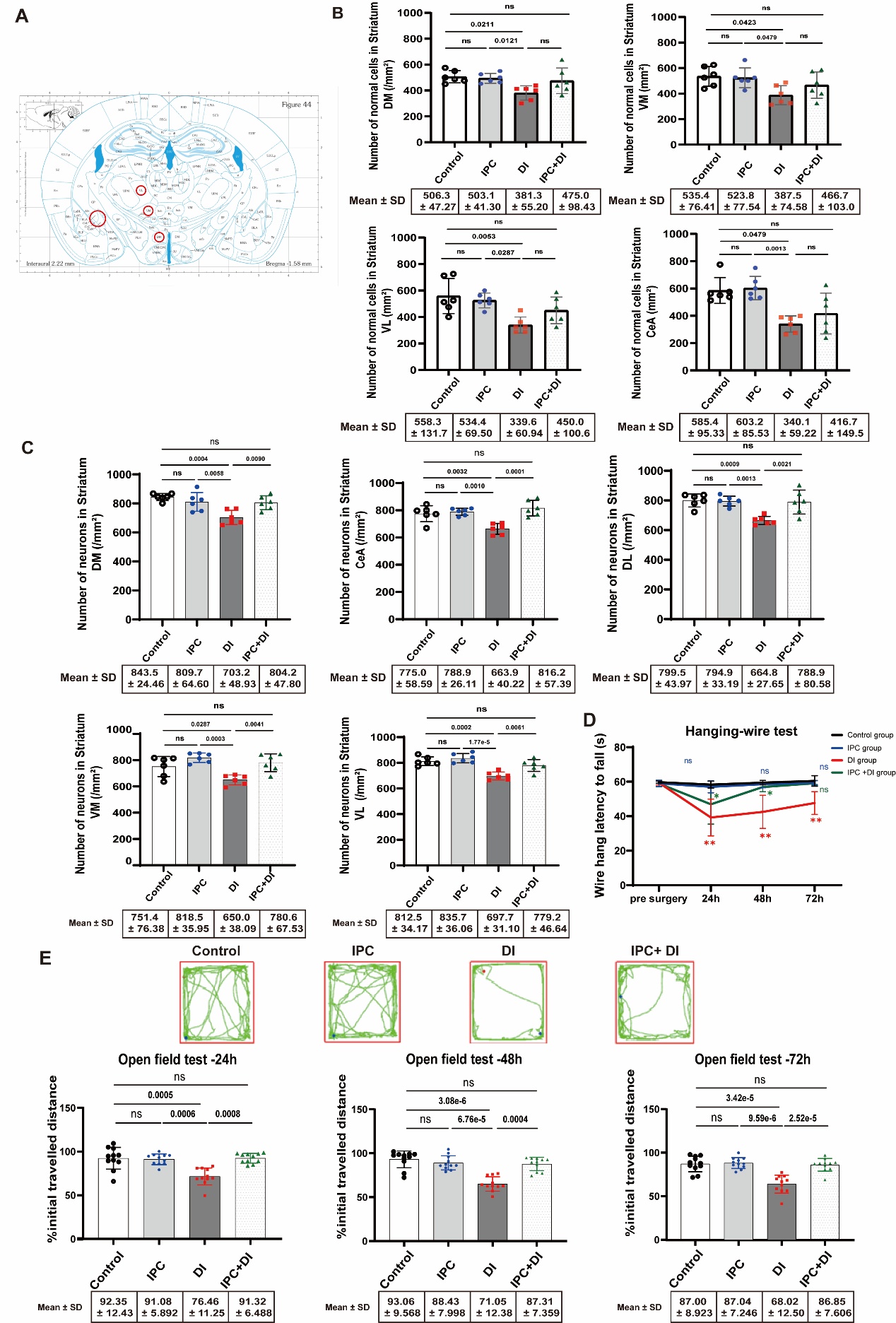


**Supplementary Figure S2. IPC-induced ischemic tolerance in the striatum. (A)** Dorsal medial (DM), ventricular medial (VM), ventricular lateral (VL), and central amygdaloid nucleus (CeA) region of striatum tested in the mouse model of global ischemia-induced ischemic tolerance. **(B)** Neuronal injury measured by Nissl staining in different striatal regions 3 days after the 17 min 2VO surgery. Error bars indicate mean ± SD, n = 6 per group. Statistical analysis was performed using one-way ANOVA coupled with Tukey's multiple comparisons test. *P* values are indicated on top of the bars; ns, no significant difference. **(C)** Neuron density in different striatal regions measured by NeuN-immunofluorescence staining. Error bars indicate mean ± SD, n = 6 per group. Statistical analysis was performed using one-way ANOVA coupled with Tukey's multiple comparisons test. *P* values are indicated on top of the bars; no significant difference. **(D)** Hanging-wire test conducted at 24h, 48h, and 72h after the 17 min 2VO surgery. Error bars indicate mean ± SD, n = 8 per group. Statistical analysis was performed using two-way ANOVA coupled with Dunnett's multiple comparisons test. *P* values are indicated on top of the bars; ns, no significant difference. **(E)** Representative tracks of the Open field test determined at 48h time point (upper panel). Quantification results of the Open field test at 24h, 48h, and 72h time points. Error bars indicate mean ± SD, n = 11. Statistical analysis was performed using one-way ANOVA coupled with Tukey's multiple comparisons test. *P* values are indicated on top of the bars; ns, no significant difference.


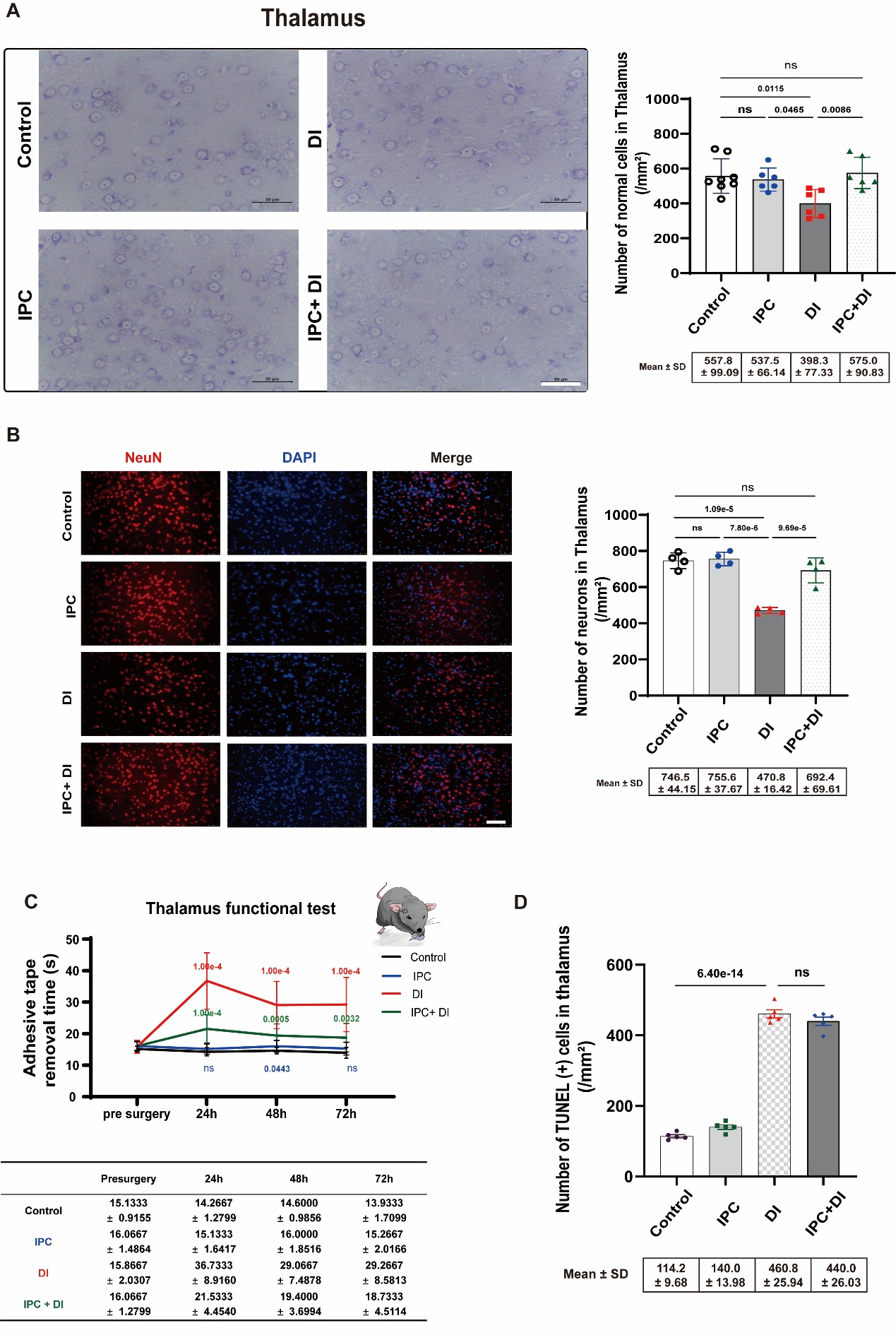


**Supplementary Figure S3. IPC induces ischemic tolerance in the thalamus. (A)** Representative images showing the neuronal injury measured by Nissl staining in the thalamus region 3 days after the 17 min 2VO surgery. Scale bar=50 μm. Right panel shows the quantification results. Error bars indicate mean ± SD, n = 8 per group. Statistical analysis was performed using one-way ANOVA coupled with Tukey's multiple comparisons test. *P* values are shown; ns, no significant difference. **(B)** Neuron density in different striatal regions measured by NeuN-immunofluorescence staining. Scale bar=50 μm. Lower panel shows the quantification results. Error bars indicate mean ± SD, n = 4 per group. Statistical analysis was performed using one-way ANOVA coupled with Tukey's multiple comparisons test. *P* values are shown; ns, no significant difference. **(C)** Striatal function was determined by the time span for the mice to remove their impaired forelimb from the adhesive tape. Data were recorded at 24h, 48h, and 72h after the 17 min 2VO surgery. Error bars indicate mean ± SD, n = 7 per group. Statistical analysis was performed using two-way ANOVA coupled with Dunnett's multiple comparisons test. *P* values for DI vs. control and IPC + DI vs. DI are indicated by blue and red numbers, respectively; ns, no significant difference. **(D)** Effect of IPC on damaging ischemia-induced injury in the thalamus determined by TUNEL-positive neurons as described in Fig. 1E. Error bars indicate mean ± SD, n = 5 per group. Statistical analysis was performed using ANOVA with Tukey’s multiple comparison test. *P* values are indicated above the bars; ns, no significant difference.

**
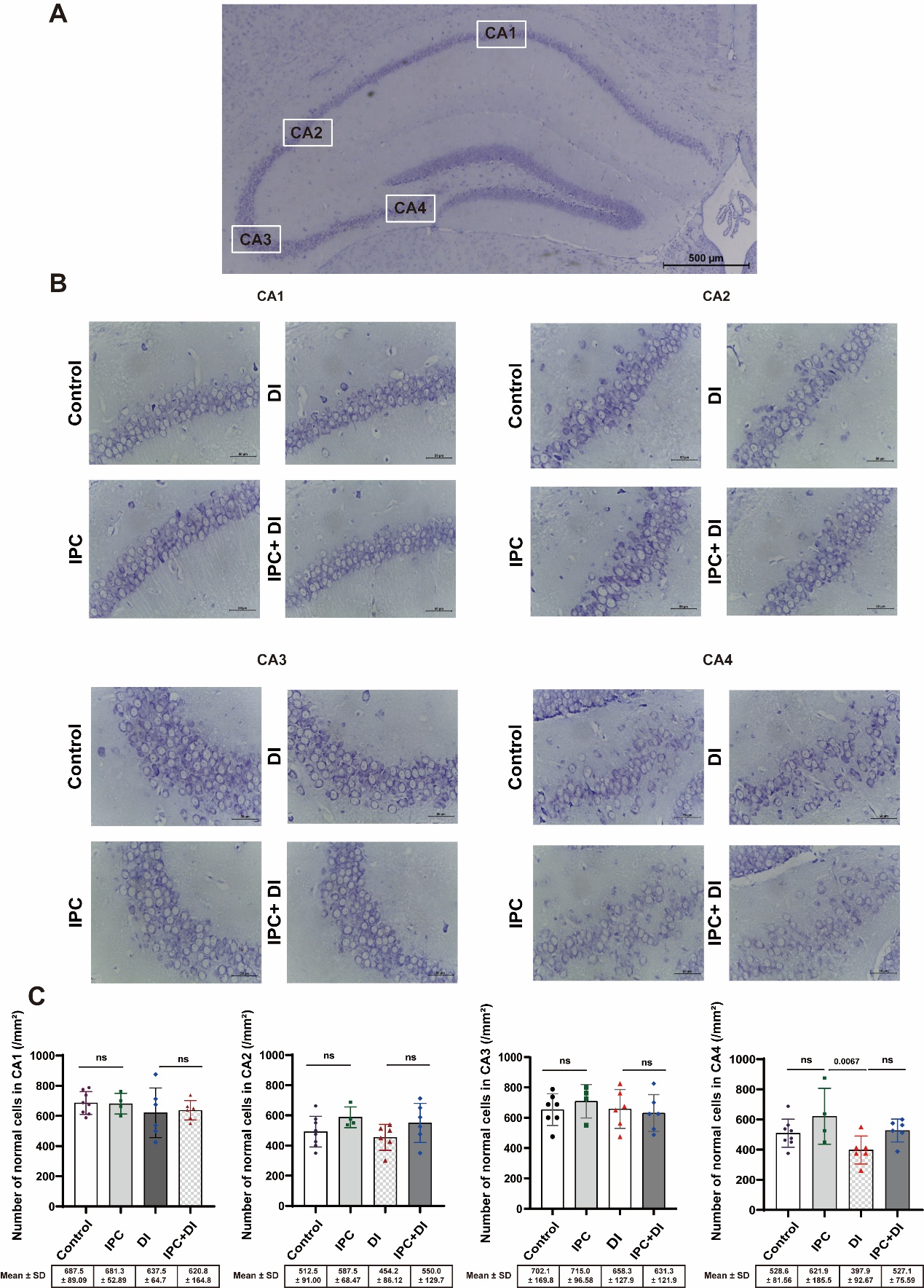
**

**Supplementary Figure S4. IPC does not induce ischemic tolerance in the hippocampus. (A)** Locations of the hippocampal regions tested in this experiment as shown by the Nissl staining. Scale bar=500 μm. **(B)** Representative images of hippocampal regions Cornu Ammonis1 (CA1) to CA4 under high magnification by Nissl staining showing neuronal damage in different regions of the hippocampus. Scale bar=50 μm. **(C)** Neuronal injury measured by Nissl staining in different hippocampal regions 3 days after the 17 min 2VO surgery. Error bars indicate ± SD, n = 4-7 per group. Statistical analysis was performed using one-way ANOVA coupled with Tukey's multiple comparisons test. *P* values are shown; ns, no significant difference.

**
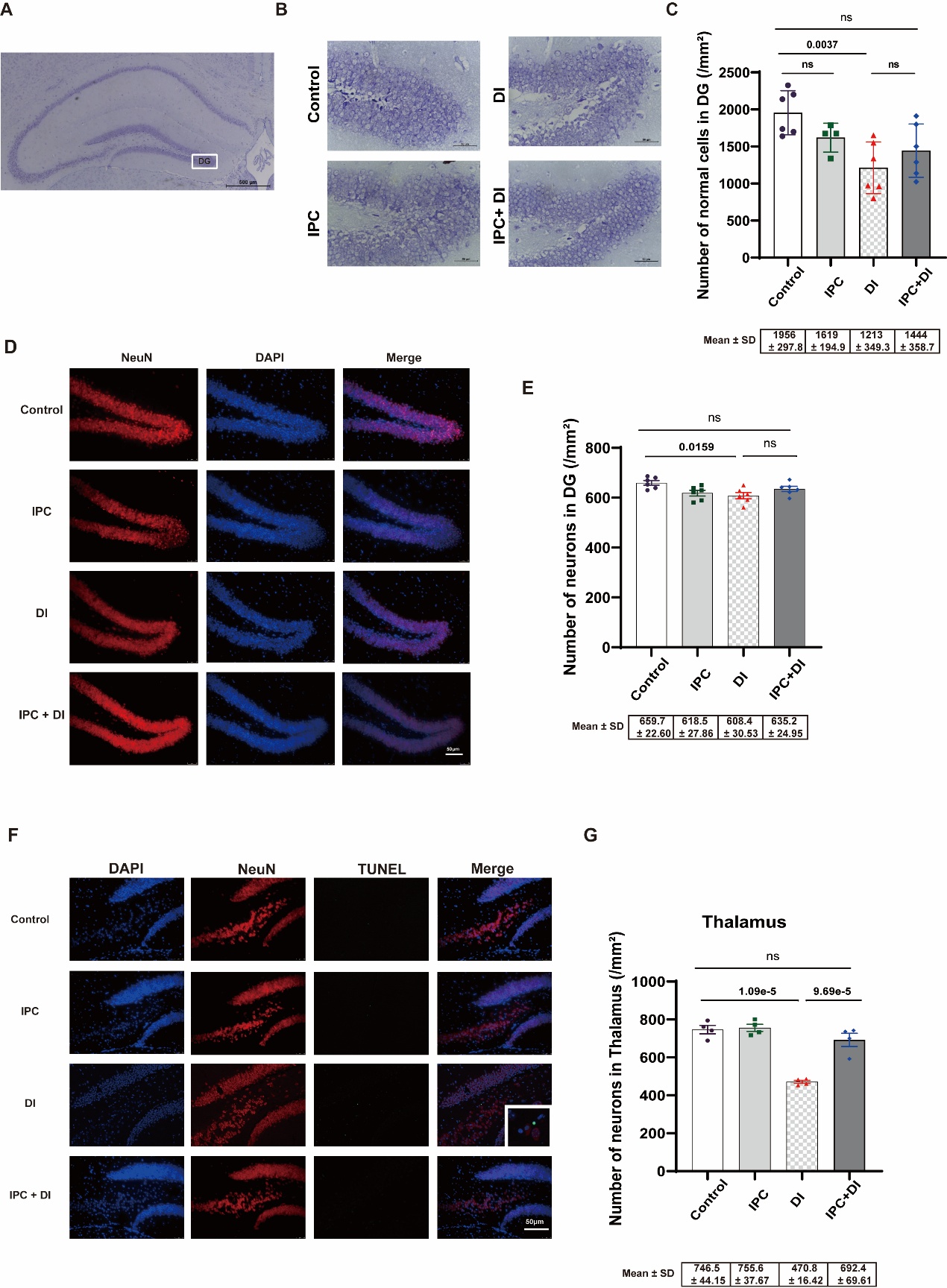
**

**Supplementary Figure S5. IPC does not induce ischemic tolerance in dentate gyrus (DG) region of the hippocampus. (A)** Locations of the hippocampal regions tested in this experiment as shown by the Nissl staining. Scale bar=500 μm. **(B)** Representative images of hippocampal DG regions under high magnification by Nissl staining showing neuronal damage in different regions of the hippocampus. Scale bar=50 μm. **(C)** Neuronal injury measured by Nissl staining in hippocampal DG regions 3 days after the 17 min 2VO surgery. Error bars indicate ± SD, n = 4-6 per group. Statistical analysis was performed using one-way ANOVA coupled with Tukey's multiple comparisons test. *P* values are shown; ns, no significant difference.

**
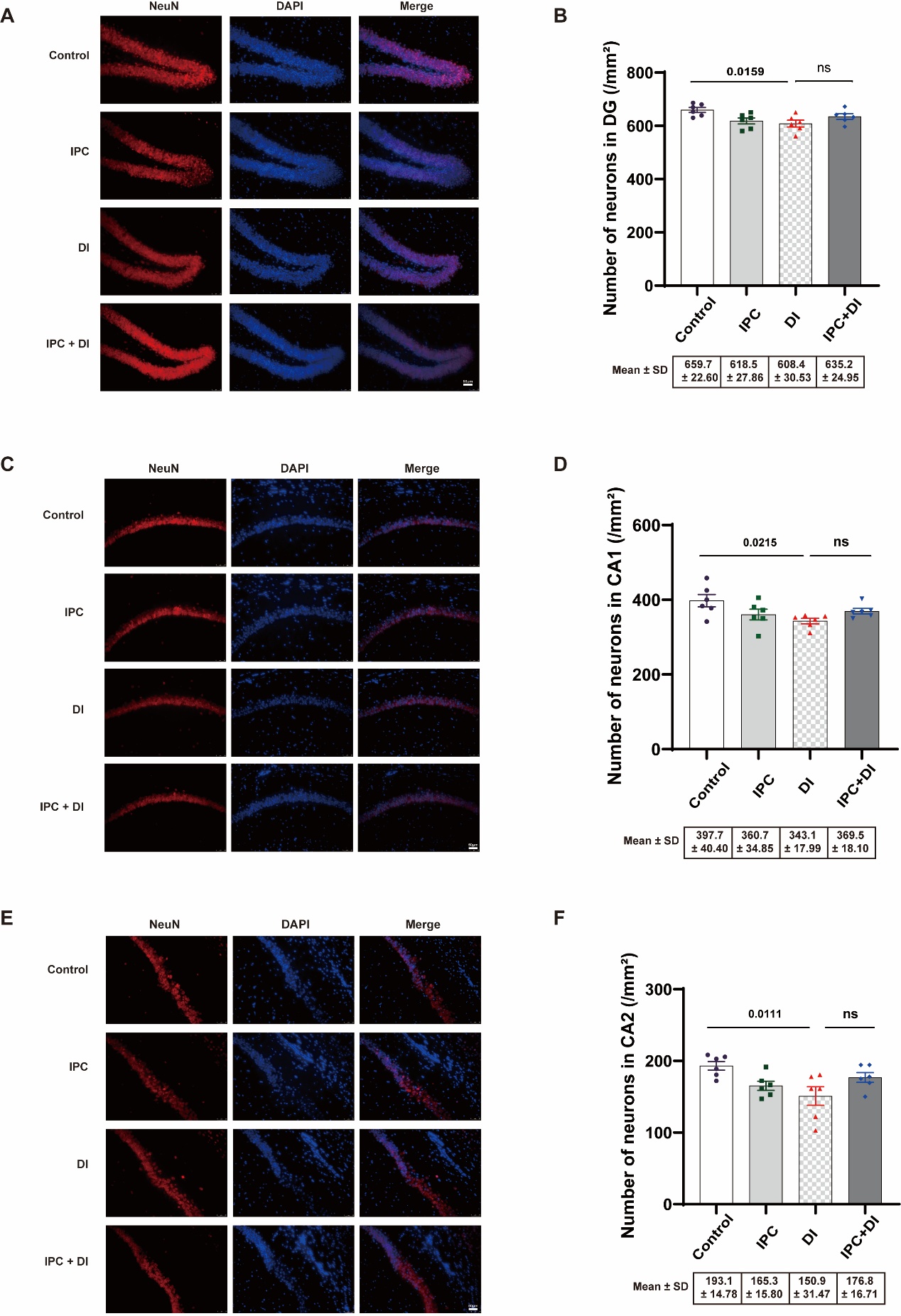
**

**Supplementary Figure S6. IPC does not induce ischemic tolerance in different regions of the hippocampus revealed by NeuN-immunofluorescence staining. (A)** Neuron density in hippocampal DG region measured by NeuN-immunofluorescence staining. Scale bar=10 μm. **(B)** Quantification results of (A). Error bars indicate mean ± SD, n = 6 per group. Statistical analysis was performed using one-way ANOVA coupled with Tukey's multiple comparisons test. *P* values are shown; ns, no significant difference. **(C)** Neuron density in hippocampal CA1 region measured by NeuN-immunofluorescence staining. Scale bar=10 μm. **(D)** Quantification results of (C). Error bars indicate mean ± SD, n = 6 per group. Statistical analysis was performed using one-way ANOVA coupled with Tukey's multiple comparisons test. *P* values are shown; ns, no significant difference. **(E)** Neuron density in hippocampal CA2 region measured by NeuN-immunofluorescence staining. Scale bar=10 μm. **(F)** Quantification results of (E). Error bars indicate mean ± SD, n = 6 per group. Statistical analysis was performed using one-way ANOVA coupled with Tukey's multiple comparisons test. *P* values are shown; ns, no significant difference.


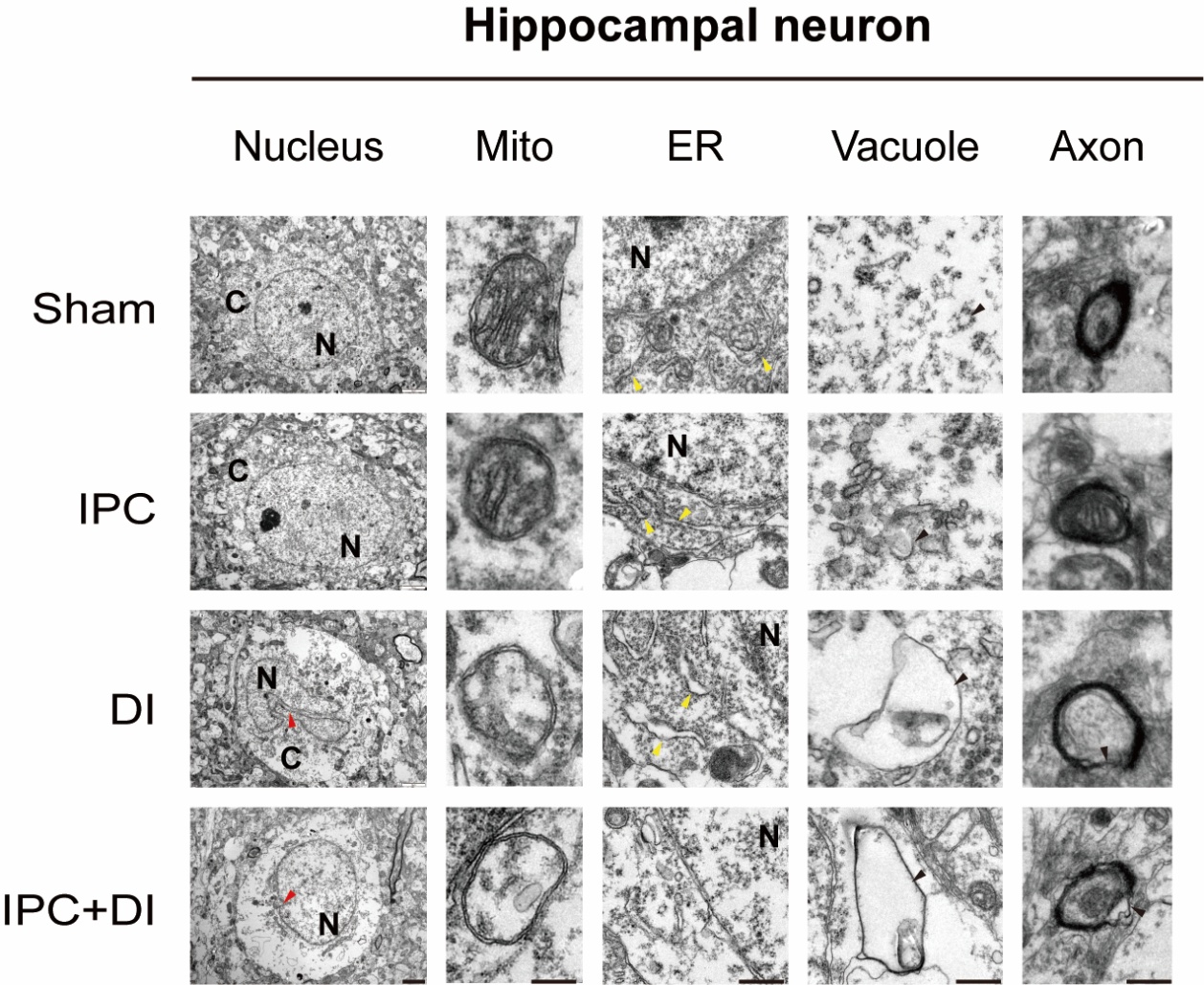


**Supplementary Figure S7. Transmission electron microscopy of the hippocampal neurons in control and different ischemic treatment groups.** The ultrastructure of major organelles including the nucleus (N), cytoplasm (C), mitochondria (Mito), endoplasmic reticulum (ER), vacuoles and axons are shown. Representative injuries in the nucleus showing deformed structure (red arrowheads), swollen mitochondria with disrupted internal cristae, distension of ER (yellow arrowheads), enlarged vacuole (blue arrowheads), cytosol with lower electron density, and axons with thinner and demyelination-like structure (black arrowheads). Scale bar: 2 μm.

**
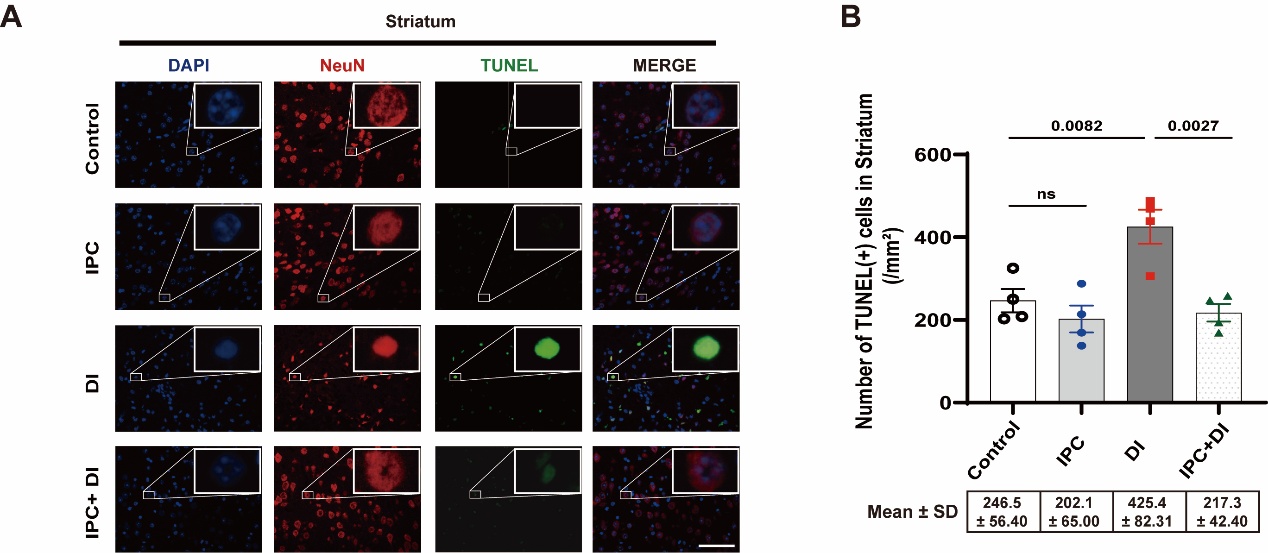
**

**Supplementary Figure S8. TUNEL immunofluorescent analysis of the striatum in the indicated mouse treatment groups.** **(A)** TUNEL immunofluorescent staining of the striatum in the indicated treatment groups. Magnified images are shown in the inset boxes. Scale bar = 100 μm. **(B)** Quantification result of the TUNEL-stained cells in (A). Error bars indicate mean ± SD, n = 4 per group. Statistical analysis was performed using ANOVA with Tukey’s multiple comparison test. *P* values are indicated above the bars; ns, no significant difference.


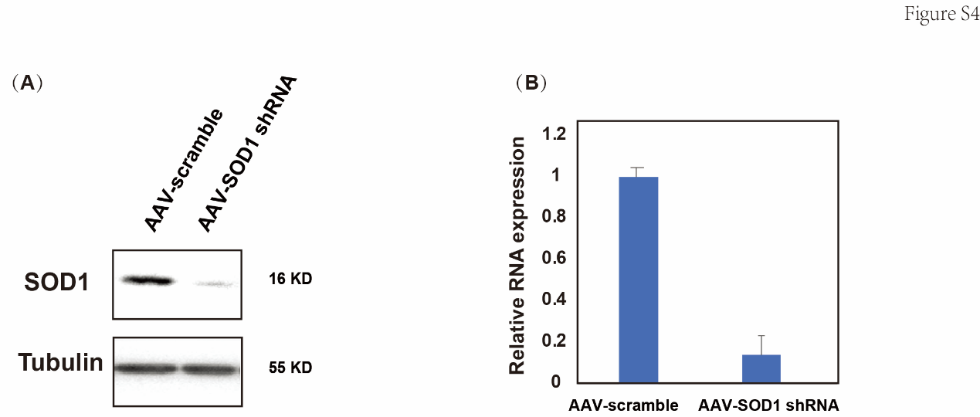


**Supplementary Figure S9. (A-B)** Knockdown efficiency mediated by the transduction of cultured cortical neurons with AAV-scramble and AAV-SOD1 shRNA as determined by Western blot (A) and RT-qPCR (B).


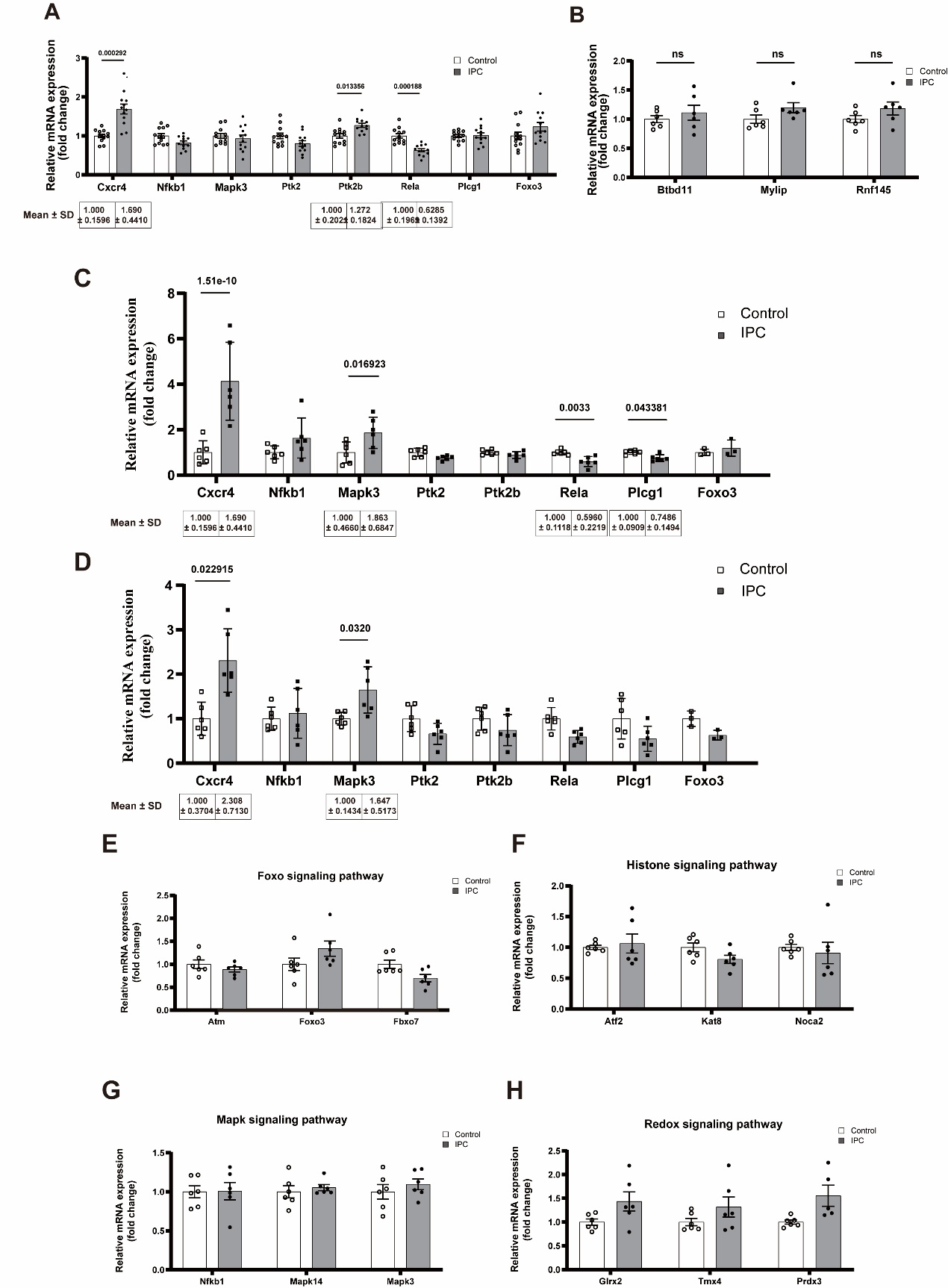


**Supplementary Figure S10. (A)** Expression of the indicated SOD1 target genes in CXCR4 pathway was determined by RT-qPCR in cultured cortical neurons treated with or without 30 min IPC followed by reperfusion for 24h. Error bars indicate mean ± SD, n = 12. Statistical analysis was performed using Student’s t-test (two-tailed). *P* values are indicated on top of the bars; ns, no significant difference. **(B)** Expression of the indicated SOD1 target genes in Ubiquitin pathway was determined by RT-qPCR in cultured cortical neurons treated with or without 30 min IPC followed by reperfusion for 24h. Error bars indicate mean ± SD, n = 6. Statistical analysis was performed using Student’s t-test (two-tailed); ns, no significant difference. **(C)** Expression of CXCR4 pathway components in the M1 cortex of mice treated with sham or IPC for 6 min 2VO as described in Fig. 1A. Error bars indicate mean ± SD; n = 6 per group. Statistical analysis was performed using Student’s t-test (two-tailed). *P* values are indicated on top of the bars. **(D)** Expression of CXCR4 pathway components in the M2 cortex of mice treated with sham or IPC for 6 min 2VO as described in Fig. 1A. Error bars indicate mean ± SD; n=6. Statistical analysis was performed using Student’s t-test (two-tailed). *P* values are indicated on top of the bars. **(E-H)** Expression of the indicated SOD1 target genes in Foxo signaling pathway (E), Histone signaling pathway (F), Mapk signaling pathway (G) and Redox signaling pathway (H) was determined by RT-qPCR in cultured cortical neurons treated with or without 30 min IPC followed by reperfusion for 24h. Error bars indicate mean ± SD, n = 6; Statistical analysis was performed using Student’s t-test (two-tailed). No significant differences between control and IPC groups were detected in all the indicated genes.

**Supplementary Table S1. Sequences of oligonucleotides**

| Gene name | Oligonucleotides | |
| --- | --- | --- |
| Nfkb1 |  | |
| forward primer | GGAGGCATGTTCGGTAGTGG | |
| reverse primer | CCCTGCGTTGGATTTCGTG | |
| Mapk3 |  | |
| forward primer | TATCAACACCACCTGCGACC | |
| reverse primer | GGATTTGGTGTAGCCCTTGGA | |
| Ptk2 |  | |
| forward primer | GAGTACGTCCCTATGGTGAAGG | |
| reverse primer | CTCGATCTCTCGATGAGTGCT | |
| Ptk2b |  | |
| forward primer | TGAGCCCTTGAGCCGTGTA | |
| reverse primer | AGCTTGAAGTTCTTCCCTGGG | |
| Rela |  | |
| forward primer | AGGCTTCTGGGCCTTATGTG | |
| reverse primer | TGCTTCTCTCGCCAGGAATAC | |
| Plcg1 |  | |
| forward primer | ATCCAGCAGTCCTAGAGCCTG | |
| reverse primer | GGATGGCGATCTGACAAGC | |
| Foxo3 |  | |
| forward primer | CTGGGGGAACCTGTCCTATG | |
| reverse primer | TCATTCTGAACGCGCATGAAG | |
| Btbd11  forward primer  reverse primer  Mylip | CTCAGGAGTTTCCAGGCCCTT  TCCATAGAAAAGCTGCGGCG | |
| forward primer | ATGCTGTGCTATGTGACGAGG | |
| reverse primer  Rnf145  forward primer  reverse primer  Prdx3  forward primer  reverse primer | | TCGATGATCCCTAGACGCC  ATGGCTGCGAAGGAGAAACTG  AAAGAGGGTTGTTGTTGAGGC  AATGGTGTCGTCAAGCACCT  ACTTGGCTTGATCGTAGGGG |
| CXCR4 | |  |
| forward primer | | GAAGTGGGGTCTGGAGACTAT |
| reverse primer  Stat3  forward primer  reverse primer  GAPDH  forward primer  reverse primer | | TTGCCGACTATGCCAGTCAAG  CAATACCATTGACCTGCCGAT  GAGCGACTCAAACTGCCCT  ACAGCAACAGGGTGGTGGAC  TTTGAGGGTGCAGCGAACTT |
